# Development and validation of a prediction model for cardiovascular–kidney–metabolic syndrome progression: a multicenter study

**DOI:** 10.3389/fnut.2026.1783262

**Published:** 2026-03-19

**Authors:** Jiawen Tu, Fangzheng Chen, Wenyue Yan, Zhiyang Xu, Yiyang Zhan, Xilan Yang

**Affiliations:** 1 Department of Geriatric Medicine, The First Affiliated Hospital of Nanjing Medical University, Nanjing, China; 2 Department of Human Resources, The Fourth Affiliated Hospital of Nanjing Medical University, Nanjing, Jiangsu, China; 3 Department of General Practice, The Fourth Affiliated Hospital of Nanjing Medical University, Nanjing, Jiangsu, China; 4 Department of Geriatric Medicine, Changzhou Geriatric Hospital Affiliated to Soochow University, Changzhou, Jiangsu, China

**Keywords:** cardiovascular–kidney–metabolic syndrome, liver metabolism axis, real-world study, risk prediction model, temporal validation

## Abstract

**Background:**

Dysfunction of multiple systems significantly contributes to cardiovascular–kidney–metabolic syndrome (CKM). We aimed to use routine indicators to predict progression to CKM and to examine the influence of the liver metabolic axis on CKM progression.

**Methods:**

This study was a multicenter retrospective study. We developed a model using new lipid-metabolism indicators to predict progression to CKM. Model performance was evaluated using discrimination, calibration, and decision curve analysis (DCA). Further studies on patients with diabetes and metabolic dysfunction-associated steatotic liver disease (MASLD) explored the link to CKM progression.

**Results:**

A total of 21,026 participants with CKM stages 1 or 2 at baseline. The model demonstrated robust discrimination, with AUCs of 0.718 (95% CI 0.704–0.730) in the 2016–2019 cohort, 0.727 (95% CI 0.714–0.740) in the 2020 cohort, and 0.747 (95% CI 0.711–0.777) in the 2021 validation cohort. The predicted-risk quartiles were 1.8, 7.6, 11.4, and 24.9%, respectively. Subgroup analyses confirmed stable discrimination across clinical subgroups and different centers. Exploratory analyses revealed that individuals with diabetes and MASLD had the highest risk of CKM progression (odds ratio [OR] 2.13, 95% CI 1.89–2.40).

**Conclusion:**

We developed a reliable model that identifies individuals at risk of progressing to CKM in the real world. Our results also suggest the liver metabolism axis may be crucial in CKM deterioration.

## Introduction

Cardiovascular–Kidney–Metabolic syndrome (CKM) is a systemic disease proposed by the American Heart Association (AHA) in 2023 that involves complex pathophysiological connections among obesity, diabetes, chronic kidney disease (CKD), and cardiovascular disease (CVD) (1). CKM is usually insidious and causes gradual multiorgan damage. By the time it reaches stage 4, the condition often deteriorates sharply. It is difficult to reverse (2), which makes early prediction and screening of high-risk CKM populations a key challenge in chronic disease prevention (3).

Previous studies have utilized public databases to predict the long-term prognosis of CKM patients, such as all-cause mortality, cardiovascular mortality, and stroke risk (4–6). Some of these studies used novel composite indicators related to metabolism or inflammation, such as the atherogenic index of plasma (AIP), the triglyceride–glucose index (TyG), or the oxidative balance score (OBS). They confirmed significant correlations with disease outcomes in patients with CKM syndrome (5, 7, 8). These studies have mainly focused on analyzing the relationships between “single or a few indicators—event outcomes.” Therefore, achieving precise prediction of the overall progression risk of CKM syndrome in real-world settings remains a gap in current research.

This study relies on multicenter health examination data and uses lipid-dominant indicators to construct and validate a CKM progression prediction model over time. For the first time, we have achieved time-based validation of risk prediction for CKM progression in real-world health examination settings, offering new tools and evidence for the early detection of high-risk individuals and targeted interventions.

## Methods

### Study design and data source

The study is a multicenter retrospective cohort analysis that adopts a temporal validation design (9). This study was approved by the Ethics Committee of the Public Health Service Center of Jiangbei New Area, Nanjing (Approval No. SFY20251104-K230). The data were obtained from the integrated Resident Health Examination Information System, which aggregates standardized health screening data from multiple hospitals and community health centers across the city and surrounding areas. All data used in this study were fully anonymized secondary data provided by the health management department for research purposes. The requirement for informed consent was waived by the ethics committee because no identifiable personal information was involved. No additional intervention measures were administered to the participants.

### Study population

**Figure 1:**
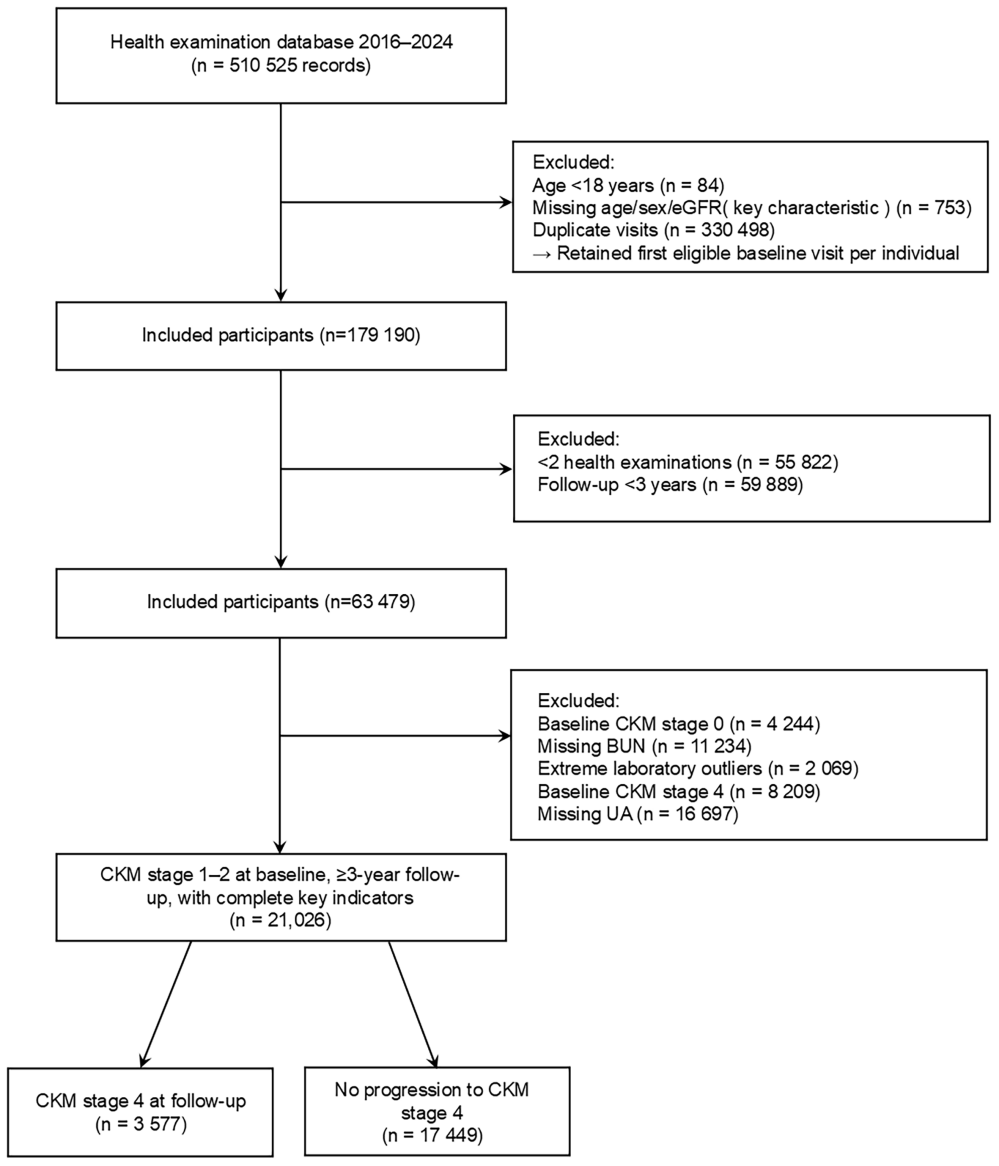
Flow diagram of participant selection. The flowchart illustrates the inclusion and exclusion criteria used to move from the original database (*n* = 510525) to the final analytic cohort (*n* = 21,026). Individuals with missing key variables, duplicates, baseline CKM stages 0 or 4, or follow-up <3 years were excluded.

The original database contained 510,525 health examination records from 2016 to 2024. After excluding individuals aged <18 years, those with missing key characteristics, duplicate visits, and follow-up <3 years, baseline CKM stage 0 or 4, missing blood urea nitrogen (BUN) or uric acid (UA), and extreme laboratory outliers, a total of 21,026 participants with CKM stage 1–2 at baseline and complete data were included in the final analysis. Accordingly, the prediction model was developed and validated for individuals with CKM stage 1–2 at baseline who had at least 3 years of follow-up, representing the intended target population for early CKM progression risk assessment. Among them, 3,577 (17.0%) progressed to CKM stage 4 during follow-up, while 17,449 (83.0%) did not. The detailed participant selection process is shown in Figure 1.

### Definitions of CKM staging, outcomes, and predictors

Progression to CKM stage 4 was defined as the occurrence of clinical cardiovascular disease (CVD) and/or advanced chronic kidney disease (CKD) at follow-up examinations, in accordance with the CKM staging framework (1). The eGFR was determined using the CKD-EPI (Chronic Kidney Disease Epidemiology Collaboration) equation, and advanced CKD was operationalized primarily using eGFR thresholds (<60 mL/min/1.73 m^2^) (10). Clinical CVD was ascertained from physician-recorded, standardized diagnoses in the regional health examination information system and medical history fields. All participating centers use the same standardized electronic platform, in which diagnoses are entered by clinicians during routine examinations based on patients’ reported medical history and clinical assessment. Diagnoses are selected from predefined diagnostic categories. Recorded CVD diagnoses included coronary artery disease, angina, myocardial infarction, heart failure, atrial fibrillation, transient ischemic attack, ischemic stroke, intracerebral hemorrhage, subarachnoid hemorrhage, peripheral arterial disease, and prior coronary revascularization procedures.

The study outcome was defined as being observed at CKM stage 4 during the follow-up examination conducted at least 3 years after baseline. Since the disease outcome was determined during routine health check-ups rather than through continuous monitoring, it was impossible to precisely observe the time of disease progression, and there was an interval censoring between the two check-ups. Therefore, our endpoint was “detecting stage 4 disease in the next available examination with an interval of ≥3 years,” rather than the exact time of disease occurrence. Candidate predictive indicators are all variables routinely collected during baseline physical examinations, primarily including demographic characteristics, such as age and sex; medical history and metabolic comorbidities, such as hypertension, diabetes, and metabolic dysfunction-associated steatotic liver disease (MASLD); and physical measurements, such as body mass index (BMI), weight-adjusted waist index (WWI). Blood pressure-related indicators, such as mean arterial pressure (MAP) and pulse pressure (PP), and renal function, such as eGFR, BUN, UA, and its creatinine ratio. Lipids and derived ratios, such as high-density lipoprotein cholesterol (HDL-C), low-density lipoprotein cholesterol (LDL-C), remnant cholesterol (RC), AIP, non-HDL-C/HDL-C ratio (NHHR), and the triglyceride (TG)/HDL-C ratio, glucose metabolism, and comprehensive metabolic indicators, such as fasting blood glucose (FBG) and TyG, and inflammation and hematology indicators, such as white blood cell count (WBC), hemoglobin (Hb), and platelet count (PLT). We systematically examined missing data across three cohorts and included only variables with complete baseline data in the main model development. To assess the potential selection bias introduced by this method, the baseline characteristics of the included participants were compared with those of participants excluded due to missing data (Supplementary Table S1). The standardized mean difference (SMD) was used to quantify the effect size, with |SMD| < 0.10 indicating a negligible difference. Missing laboratory data occurred mainly because some centers or years did not routinely measure these tests, therefore, the missing-data mechanism cannot be formally determined from the data. To ensure robustness of the complete-case approach under conservative assumptions about missing predictors, we conducted an extreme-case sensitivity analysis (5th/95th percentile imputation) in the development cohort and found that discrimination and calibration in the fixed 2021 temporal validation cohort were essentially unchanged, indicating stable model performance despite missingness (Supplementary Table S2).

### Statistical analysis

For all continuous variables, we calculated the 0.2 and 99.8 percentiles and excluded participants with values exceeding these thresholds to reduce the influence of extreme outliers on the model (Supplementary Table S3). We compared the standardized mean differences (SMDs) of the continuous variables to ensure that no significant selection bias would be introduced (11). All the data preprocessing parameters were applied to the training set (2016–2019).

To control for multicollinearity while maintaining clinical rationality, we performed clustering and variable simplification before modeling. In addition to the key baseline variables, the other indicators were grouped by clinical attributes. We first calculated Spearman correlation coefficients among the candidate variables and then used hierarchical clustering to group highly correlated variables (|*ρ*| ≥ 0.90) into distinct clusters. Each cluster selects representative variables and adds them to the candidate pool. Candidate predictors were grouped into clinically coherent pathophysiological domains (anthropometric/adiposity, blood pressure/hemodynamic load, renal–uric acid metabolism, lipid dysregulation, glycemic/insulin resistance, and inflammatory/hematological status). Within each domain, correlation-based clustering was applied to reduce redundancy while retaining representative features capturing the dominant signal of each biological pathway (Supplementary Table S4).

The final model was trained using elastic net–regularized logistic regression (*α* = 0.5). The regularization parameter (*λ*) was tuned by 10-fold cross-validation with AUC as the optimization metric (12). To control model complexity, we restricted candidate solutions along the *λ* path to those with no more than 12 non-zero coefficients (excluding the intercept). Among these candidates, the model achieving the highest cross-validated AUC was selected as the final model (*λ* = 0.00497). Sample size adequacy was evaluated using the events-per-variable (EPV) criterion, following recommendations for prediction model development (13). In the training cohort (2016–2019), there were 1,767 CKM stage 4 events. The final model retained 9 predictors, yielding an EPV of 196.3, which substantially exceeds commonly recommended thresholds for penalized logistic regression and supports the stability of model estimation. In the cohort, continuous variables were standardized using z scores calculated from the mean and standard deviation. The final model coefficients (intercept and all retained predictors) and the standardization parameters are provided in Supplementary Table S5.

Model performance was assessed by the AUC (2,000 bootstrap 95% CIs), calibration intercept/slope, Brier score, and locally estimated scatterplot smoothing (LOESS) calibration plots. Clinical utility was examined using decision curve analysis (DCA) (net benefit thresholds of 0–30% vs. treat-all/none strategies) (14, 15), and risk stratification was assessed by comparing observed event rates across predicted risk quartiles. No formal statistical tests were conducted to compare the AUC values among the subgroups in the subgroup analysis; the AUC results were only interpreted descriptively.

Baseline characteristics across the training, validation, and test cohorts were compared using the Kruskal–Wallis test for continuous variables and the *χ*^2^ test for categorical variables. Socioeconomic variables were described descriptively without formal hypothesis testing for between-cohort differences.

This study conducted a stratified exploratory analysis of diabetes and MASLD. The diagnosis of MASLD is based on ultrasound results, as well as metabolic and drinking conditions (16). Multivariate logistic regression models were used to estimate odds ratios (ORs) and 95% confidence intervals for progression to CKM stage 4 within each group.

All statistical analyses were conducted in R (version 4.5.0). The main R packages included glmnet (penalized logistic regression), pROC (ROC curves and AUC), boot (bootstrapping), ggplot2 (visualization), etc. All statistical tests were two-tailed, and a *p*-value less than 0.05 was considered statistically significant.

## Results

### Baseline characteristics

A total of 21,026 participants were included in the final analysis (57.0% male; mean ± standard deviation [SD] age, 65.6 ± 7.2 years). The participants were classified into three time-based cohorts. The patients’ baseline characteristics are summarized in Table 1. The sex distribution remained consistent at 57.0% male overall (*p* = 0.005). Additionally, LDL-C increased from 111.3 to 117.2 mg/dL, and total cholesterol (TC) increased from 192.3 to 198.6 mg/dL. The proportion of individuals with hypertension decreased from 65.8 to 56.1%, whereas the prevalence of diabetes mellitus decreased from 27.1 to 19.8% (*p* < 0.001 for both). BMI (25.0 ± 3.0 kg/m^2^, *p* = 0.720) and the distribution of baseline CKM stages (*p* = 0.178) remained consistent. The median intervals between baseline and the next available examination were 4.8 (4.4–5.0), 3.8 (3.7–4.0), and 3.1 (3.1–3.4) years in the development, calibration, and validation cohorts, respectively (*p* < 0.001).

**Table 1:** Baseline characteristics of the temporal cohorts.

Characteristic	Overall *N* = 21,026	TRAIN *N* = 7,831	VAL *N* = 11,235	TEST *N* = 1,960	*p* value
Demographics					
Age, mean (SD), years	65.6 ± 7.2	66.5 ± 7.4	65.6 ± 6.9	62.2 ± 7.2	<0.001
Sex, *n* (%)					0.005
Female	9,035 (43.0)	3,449 (44.0)	4,801 (42.7)	785 (40.1)	
Male	11,991 (57.0)	4,382 (56.0)	6,434 (57.3)	1,175 (59.9)	
Lifestyle					
Smoking status, *n* (%)					0.002
No	17,618 (83.8)	6,647 (84.9)	9,320 (83.0)	1,651 (84.2)	
Yes	3,408 (16.2)	1,184 (15.1)	1,915 (17.0)	309 (15.8)	
Alcohol consumption, *n* (%)					<0.001
No	16,622 (79.1)	6,251 (79.8)	8,779 (78.1)	1,592 (81.2)	
Yes	4,404 (20.9)	1,580 (20.2)	2,456 (21.9)	368 (18.8)	
Socioeconomic/family					
Education level, *n* (%)					
≤Middle school	7,767 (36.9)	2,544 (32.5)	4,690 (41.7)	533 (27.2)	
≥Middle school	13,259 (63.1)	5,287 (67.5)	6,545 (58.3)	1,427 (72.8)	
Marital/living status, *n* (%)					
Cohabitation	18,975 (90.2)	6,891 (88.0)	10,259 (91.3)	1,825 (93.1)	
Living alone	2,051 (9.8)	940 (12.0)	976 (8.7)	135 (6.9)	
Blood pressure and adiposity					
BMI, mean (SD), kg/m^2^	25.0 ± 3.0	25.0 ± 2.9	25.0 ± 3.0	25.1 ± 3.0	0.720
Waist, mean (SD), cm	85.6 ± 8.2	85.3 ± 8.1	85.9 ± 8.2	85.5 ± 8.2	<0.001
SBP, mean (SD), mmHg	135.6 ± 16.1	135.4 ± 15.8	135.6 ± 16.1	136.7 ± 17.3	<0.001
DBP, mean (SD), mmHg	80.5 ± 9.9	79.2 ± 9.8	81.0 ± 9.8	82.9 ± 10.4	<0.001
Renal and metabolic biomarkers					
eGFR, mean (SD), mL/min/1.73 m^2^	92.0 ± 11.6	91.2 ± 12.0	92.1 ± 11.2	93.8 ± 11.3	<0.001
Scr, mean (SD), mg/dL	0.8 ± 0.2	0.8 ± 0.2	0.8 ± 0.2	0.8 ± 0.2	0.024
Bun, mean (SD), mg/dL	5.6 ± 1.2	5.6 ± 1.2	5.6 ± 1.2	5.4 ± 1.1	<0.001
FBG, mean (SD), mmol/L	5.9 ± 1.6	5.9 ± 1.6	6.0 ± 1.5	5.9 ± 1.4	0.001
TG, mean (SD), mg/dL	153.6 ± 89.5	152.6 ± 84.0	155.1 ± 93.0	149.5 ± 90.3	<0.001
HDL-C, mean (SD), mg/dL	53.4 ± 12.5	53.3 ± 13.1	53.6 ± 12.1	52.9 ± 12.7	<0.001
LDL-C, mean (SD), mg/dL	115.3 ± 32.3	111.3 ± 34.3	117.8 ± 30.6	117.2 ± 32.2	<0.001
TC, mean (SD), mg/dL	193.8 ± 37.8	192.3 ± 39.0	194.0 ± 36.6	198.6 ± 38.7	<0.001
Comorbidities and baseline status					
Hypertension, *n* (%)					<0.001
No	7,941 (37.8)	2,677 (34.2)	4,403 (39.2)	861 (43.9)	
Yes	13,085 (62.2)	5,154 (65.8)	6,832 (60.8)	1,099 (56.1)	
Diabetes, *n* (%)					<0.001
No	15,895 (75.6)	5,711 (72.9)	8,612 (76.7)	1,572 (80.2)	
Yes	5,131 (24.4)	2,120 (27.1)	2,623 (23.3)	388 (19.8)	
Baseline CKM stage, *n* (%)					0.178
CKM stage 1	873 (4.2)	344 (4.4)	440 (3.9)	89 (4.5)	
CKM stage 2	20,153 (95.8)	7,487 (95.6)	10,795 (96.1)	1,871 (95.5)	
Follow-up interval, years, median (IQR)	3.9 (3.6–4.6)	4.8 (4.4–5.0)	3.8 (3.7–4.0)	3.1 (3.1–3.4)	<0.001

Continuous variables are presented as the means ± SDs and were compared via the Kruskal–Wallis test. Categorical variables are presented as *n* (%) and were compared via the *χ*^2^ test. Socioeconomic variables were described without testing for between-cohort differences. BMI, body mass index; SBP, systolic blood pressure; DBP, diastolic blood pressure; eGFR, estimated glomerular filtration rate; Scr, serum creatinine; BUN, blood urea nitrogen; FBG, fasting blood glucose; TG, triglycerides; HDL-C, high-density lipoprotein cholesterol; LDL-C, low-density lipoprotein cholesterol; TC, total cholesterol; CKM, cardiovascular–kidney–metabolic; SD, standard deviation; IQR, interquartile range.

This study included a total of 120 independent medical examinations or healthcare institutions (Supplementary Table S6), including community health centers (44 institutions), hospitals (51 institutions), outpatient departments (six institutions), township health centers/health stations (four institutions), clinics (one institution), health examination centers (eight institutions), and other types of institutions (six institutions). The overall sample distribution was balanced, thereby reducing the potential influence of the central effect on the analysis results.

The composition of CKM stage 4 events in the 2016–2021 cohort revealed that approximately 74.3% of the CKM 4 events were caused solely by clinical CVD (CVD only), and 25.7% had both clinical CVD and an eGFR reduction (CVD + eGFR < 60) (Supplementary Table S7).

### Model performance and temporal stability

**Figure 2:**
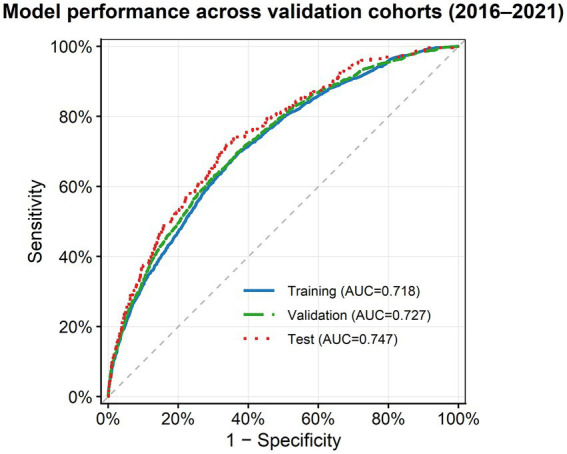
ROC curves for model performance in time-split cohorts. The ROC curves show the discrimination ability of the elastic-net model across the development (2016–2019), calibration (2020), and temporal validation (2021) cohorts. The AUCs were 0.718, 0.727, and 0.747, respectively. ROC, receiver operating characteristic; AUC, area under the receiver operating characteristic curve.

An elastic-net model was developed using routinely collected health examination parameters, and its predictive performance was systematically assessed across time-stratified cohorts. The model showed consistently robust discrimination across all temporal datasets (Figure 2; Supplementary Table S8). The AUC was 0.718 (95% CI 0.704–0.730) in the 2016–2019 development cohort, 0.727 (95% CI 0.714–0.740) in the 2020 calibration cohort, and 0.747 (95% CI 0.711–0.777) in the 2021 temporal validation cohort.

### Calibration and clinical utility

In the 2021 validation cohort, the model demonstrated overall good calibration. The LOESS-smoothed calibration curve (Supplementary Figure S1) was generally parallel to the 45° reference line, with a slight downward shift, indicating mild overall risk overestimation. The calibration intercept (calibration-in-the-large) was −0.183 (95% CI −0.523 to 0.122), and the calibration slope was 1.201 (95% CI 0.994–1.398), suggesting no major systematic miscalibration across the clinically relevant range of predicted risks.

**Figure 3:**
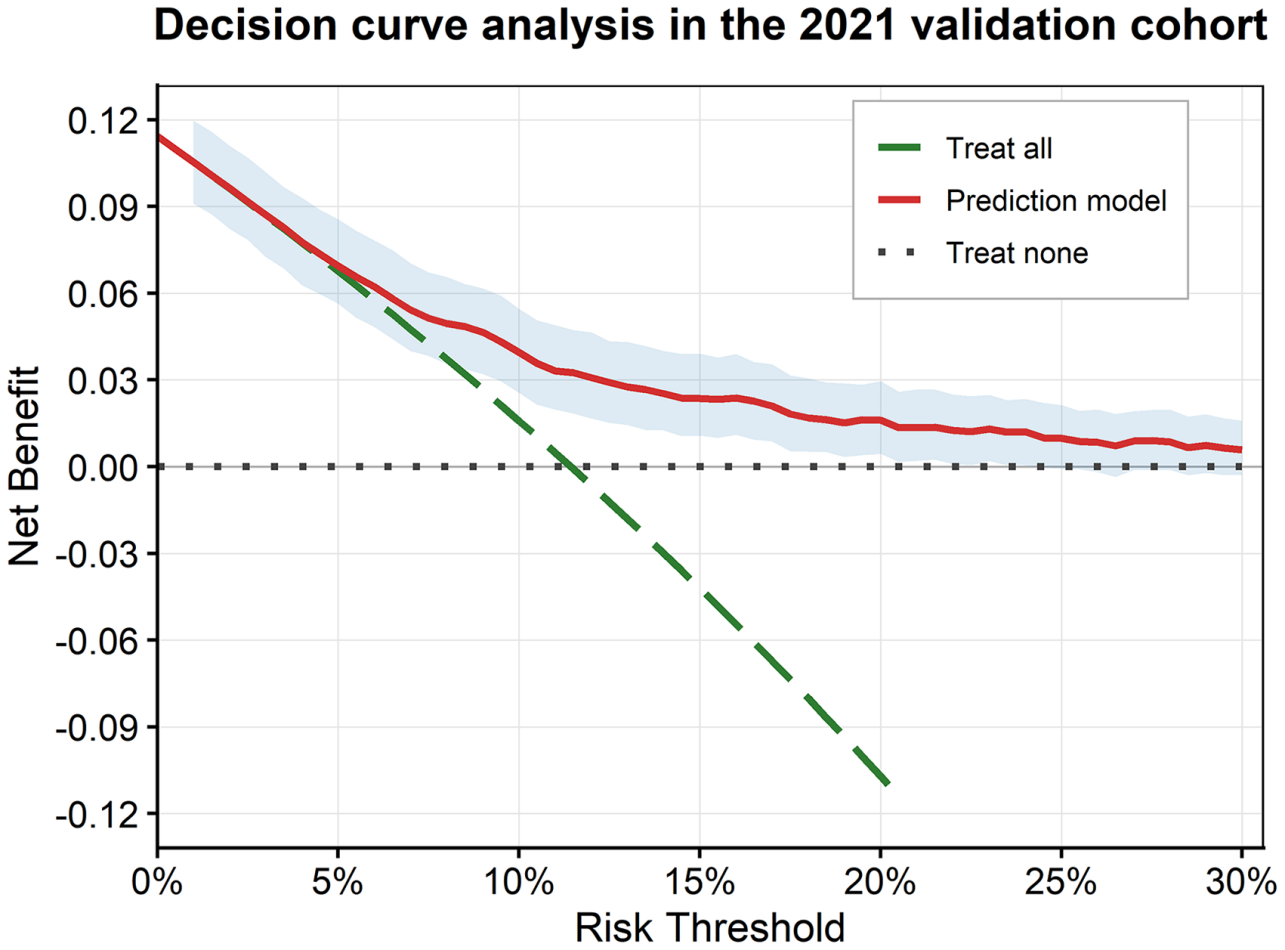
Decision curve analysis (DCA) in the 2021 temporal validation cohort. The DCA shows the net clinical benefit of using the model to guide intervention decisions across a range of threshold probabilities (0–30%). The model provided a greater net benefit than the “treat-all” or “treat-none” strategies across the clinically relevant range.

The DCA in the 2021 validation cohort (Figure 3) showed that the model provided a consistently higher net benefit than the “treat-all” and “treat-none” strategies across a range of clinically plausible threshold probabilities (approximately 5–30%). This indicates that using the model to guide risk-stratified screening or intensified follow-up could yield greater clinical benefit than non-selective strategies in real-world health examination settings. The maximum net benefit was approximately 0.14, supporting the potential clinical utility of the model in identifying individuals at high risk of CKM stage 4 progression.

### Predictors in the final model

The final model included nine continuous variables (Table 2), namely, age, AIP, eGFR, Hb, LDL-C, PP, UA, TyG, and WWI. When expressed as an increase of one standard deviation, the strongest risk gradient was for age (OR 1.84), followed by TyG (OR 1.20), AIP (OR 1.10), and WWI (OR 1.02). In contrast, higher eGFRs and hemoglobin levels are associated with lower risks (ORs of 0.77 and 0.90, respectively).

**Table 2:** Association between predictors and CKM4 (per clinical unit).

Variable	Effect (clinical unit)	OR (95% CI)
Age (years)	Per 10 years	1.84 (1.68–2.02)
AIP (index)	Per 0.25 index	1.10 (0.98–1.22)
eGFR (mL/min/1.73 m^2^)	Per 10 mL/min/1.73 m^2^	0.77 (0.73–0.81)
Hb (g/dL)	Per 1 g/dL	0.90 (0.86–0.94)
LDL-C (mg/dL)	Per 10 mg/dL	0.97 (0.95–0.99)
PP (mmHg)	Per 10 mmHg	0.99 (0.95–1.03)
UA (mg/dL)	Per 1 mg/dL	1.09 (1.05–1.14)
TyG (index)	Per 0.5 index	1.20 (1.08–1.33)
WWI (cm/√kg)	Per 0.5 index	1.04 (1.00–1.08)

AIP, atherogenic index of plasma; eGFR, estimated glomerular filtration rate; Hb, hemoglobin; LDL-C, low-density lipoprotein cholesterol; PP, pulse pressure; TyG, triglyceride–glucose index; UA, uric acid; WWI, weight-adjusted waist index; OR, odds ratio; CI, confidence interval. Definitions of derived indices: AIP = log10[TG (mg/dL)/HDL-C (mg/dL)]; TyG = ln[TG (mg/dL) × FBG (mg/dL)/2]; WWI = WC (cm)/√weight (kg). AIP and TyG are unitless indices; WWI is expressed as cm/√kg.

### Risk stratification performance

**Figure 4:**
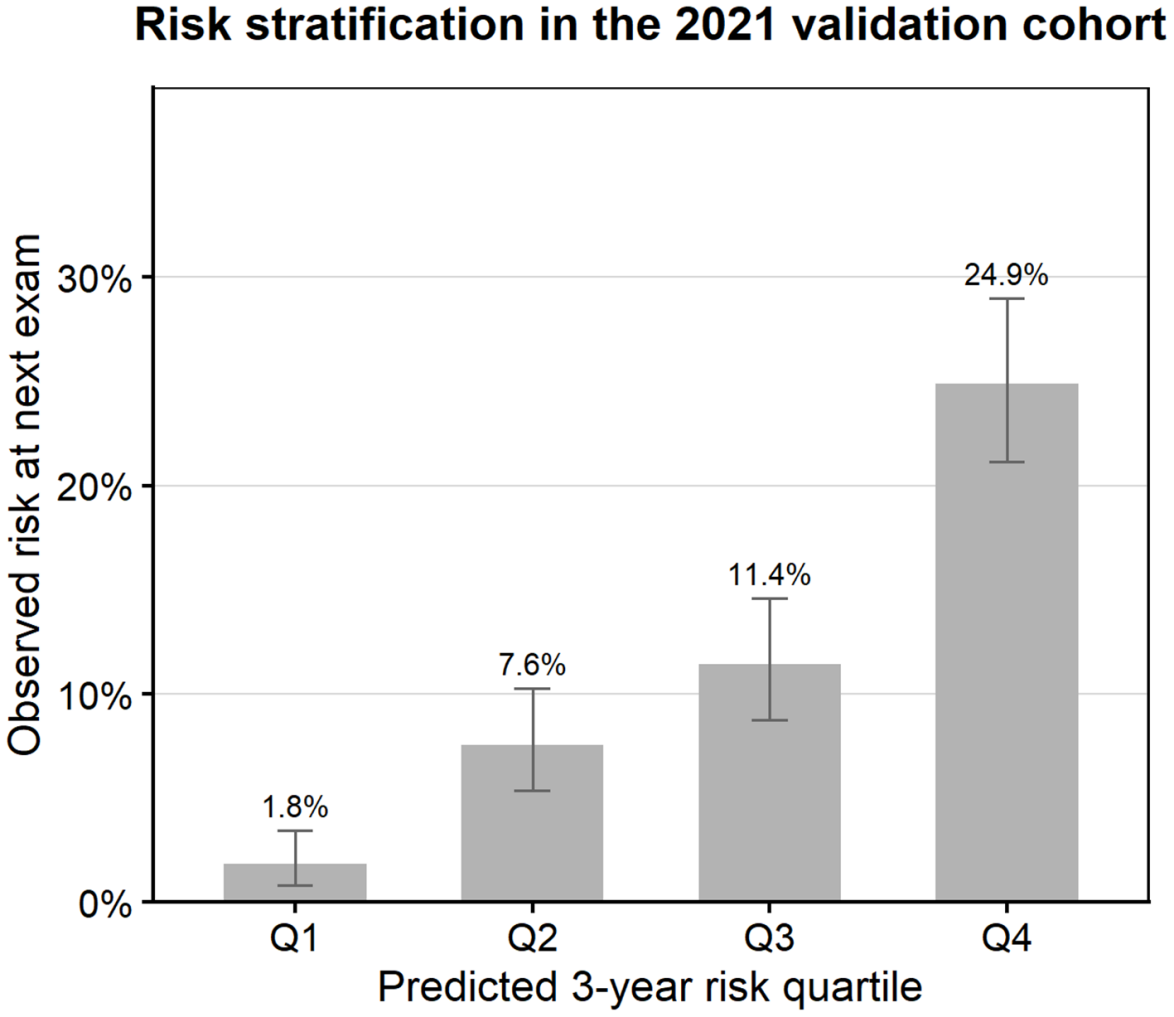
Risk stratification by quartiles of predicted risk in the 2021 temporal validation cohort. The CKM stage 4 proportions observed at the next health examination increased progressively from 1.8% (Q1) to 7.6% (Q2), 11.4% (Q3), and 24.9% (Q4), indicating effective separation of the low-, medium-, and high-risk groups.

To assess the model’s ability to distinguish risks, we divided participants in the 2021 validation cohort into four equal groups based on predicted risk scenarios (Figure 4; Supplementary Table S9). The observed proportion of CKM stage 4 cases showed a gradient: Q1 was 1.8%, Q2 was 7.6%, Q3 was 11.4%, and Q4 was 24.9% (*n* = 490 per group). The risk for individuals in the highest-risk quartile was approximately 13 times that of individuals in the lowest-risk quartile, confirming that the model could effectively classify the population.

### Nomogram for individualized risk prediction

To facilitate its clinical application, we developed a nomogram (Supplementary Figure S2) based on the final elastic network model. This tool simplifies the multivariate regression equation into a clear scoring system, allowing clinicians to estimate the probability that an individual will progress to CKM stage 4 within 3 years by summing the scores for the nine predictive indicators.

### Subgroup and sensitivity analyses

To investigate whether discrimination is related to follow-up time, we limited the analysis to the 2021 validation cohort and divided participants into three groups based on the observed distribution of follow-up time. The event rates in the follow-up groups were 9.1, 13.1, and 12.1%, respectively (Supplementary Figure S3; Supplementary Table S10). The AUCs were 0.717 (95% CI 0.645–0.790), 0.742 (95% CI 0.688–0.796), and 0.771 (95% CI 0.720–0.823), indicating that the model’s discrimination performance was relatively stable among individuals with different follow-up durations.

In the sensitivity analysis, we examined whether model performance was affected by individuals whose baseline renal function was near the CKM diagnostic threshold. In the 2021 time-validation cohort, participants with baseline eGFRs < 65 mL/min/1.73 m^2^ were excluded, and only those with relatively good renal function were retained for analysis (*n* = 1,920; events = 209). The model’s AUC was 0.742 (95% CI 0.707–0.777), which was almost consistent with that of the overall validation cohort (Supplementary Table S11).

A multicenter analysis conducted in the 2021 time validation cohort revealed that the model’s discriminative ability was generally consistent across centers with sufficient event numbers, and most 95% confidence intervals overlapped (Supplementary Figure S4; Supplementary Table S12). Several centers with tiny sample sizes had only 1–2 events, resulting in high AUC values but wide confidence intervals and insufficient statistical stability. Center-specific calibration analysis further showed that, among centers with ≥15 events, calibration intercepts and slopes had 95% confidence intervals containing the ideal values (0 and 1), and expected/observed ratios were close to 1, indicating no evidence of systematic miscalibration in centers with reliable sample sizes (Supplementary Table S13).

Compared with a parsimonious reference model including only age, sex, eGFR, and diabetes status (AUC in the 2021 test cohort = 0.674, 95% CI 0.632–0.712), the final multivariable model showed improved discrimination (AUC = 0.749, 95% CI 0.713–0.780), supporting its incremental predictive value beyond basic clinical predictors. This incremental value was further supported by reclassification metrics in the 2021 temporal validation cohort, with a significant improvement in category-free net reclassification improvement (continuous NRI = 0.240, 95% CI 0.106–0.377) and integrated discrimination improvement (IDI = 0.0087, 95% CI 0.0009–0.0167) (Supplementary Figure S6).

The subgroup analysis in the 2021 temporal validation cohort showed generally consistent discrimination across clinically relevant subgroups (Supplementary Figure S5; Supplementary Table S14). However, several subgroups exhibited deviations in calibration, including participants younger than 60 years (CITL −0.704; slope 1.400), those with an estimated glomerular filtration rate (eGFR) of 75–<90 mL/min/1.73 m^2^ (CITL −0.631; slope 1.568), individuals without MASLD (CITL −0.647), and those with baseline CKM stage 1 (AUC 0.764; CITL −1.253; slope 2.029).

The CKM stage 1 subgroup in the 2021 cohort included 89 participants with only 3 outcome events. With such sparse event counts, subgroup-specific discrimination and calibration estimates are inherently unstable and highly sensitive to small fluctuations in predicted probabilities. Therefore, the observed AUC value in this subgroup (0.764) should be interpreted cautiously and does not necessarily reflect reliable subgroup discrimination.

Given these findings, we conducted post-hoc global recalibration by fitting a logistic recalibration model in the 2021 cohort (logit[y] = *α* + *β*·logit[p]), yielding *α* = −0.183 and *β* = 1.201. After recalibration, calibration-in-the-large was markedly attenuated and slopes moved closer to 1 in most subgroups (e.g., Age <60: CITL −0.140; slope 1.165; No MASLD: CITL −0.151; slope 1.025; eGFR 75–<90: slope 1.305), while discrimination (AUC) remained unchanged as expected under logit-scale linear transformation (Supplementary Table S15).

To further assess the stability of model performance in CKM Stage 1 individuals, we performed an additional pooled descriptive assessment combining all available cohorts (2016–2021). The pooled dataset included 873 Stage 1 participants with 87 outcome events, providing substantially greater statistical stability than the 2021 cohort alone. In this pooled sample, discrimination remained moderate (AUC 0.676, 95% CI 0.609–0.731) and calibration was acceptable (CITL −0.244, 95% CI −0.920 to 0.435; slope 1.061, 95% CI 0.707–1.459). Because the pooled dataset includes participants from the development and earlier validation cohorts, this analysis is presented as a descriptive stability assessment rather than an independent external validation (Supplementary Table S16).

In scenario-based stress testing, perturbation of key predictors (TyG, AIP, eGFR, PP, age, WWI, Hb, LDL-C, and UA) resulted in stable discrimination and calibration slope across all scenarios, whereas calibration-in-the-large exhibited systematic negative shifts under adverse metabolic profiles, suggesting potential underestimation of absolute risk in populations with poorer metabolic control (Supplementary Table S17).

To formally quantify robustness to unmeasured confounding from medication use and lifestyle factors at the association level, we additionally conducted E-value analyses. As shown in Supplementary Table S18, the strongest predictors (age, Hb, TyG, and eGFR) yielded E-values of approximately 2.0–2.4. This indicates that an unmeasured confounder would need to be associated with both the predictor and CKM progression by a risk ratio of at least twofold to fully explain away these associations. In contrast, the remaining five predictors (AIP, UA, PP, LDL-C, and WWI) had 95% confidence intervals that included the null, resulting in CI-bound E-values of 1.00. This indicates that even modest unmeasured confounding could render these individual associations non-significant, and their contributions should be interpreted with caution.

### Exploratory analysis

In the exploratory analysis, both MASLD and diabetes were linked to a greater risk of CKM stage 4 progression (Supplementary Table S19). Compared with individuals without either condition, those with only MASLD and those with only diabetes had similar risks of progression (OR 1.75, 95% CI 1.60–1.92; OR 1.72, 95% CI 1.53–1.93; both *p* < 0.001), whereas individuals with both diabetes and MASLD presented the highest risk (OR 2.13, 95% CI 1.89–2.40; *p* < 0.001). When a multiplicative interaction term between MASLD and diabetes was introduced, the interaction was statistically significant (OR for interaction = 0.71, 95% CI 0.60–0.84; *p* for interaction < 0.001), indicating effect modification on the multiplicative scale, with a weaker incremental association of MASLD among individuals with diabetes.

To assess whether the interaction term between MASLD and diabetes could improve predictive performance, we forced it into the elastic net model. In the temporal validation cohort (2021), model performance remained essentially unchanged: the AUC was 0.747 without the interaction and 0.746 with the interaction; the Brier score was 0.0943 in both models; calibration-in-the-large (CITL) was −0.183 versus −0.182; and the calibration slope remained 1.20 in both cases. Similar findings were observed in the development cohort (AUC 0.718 vs. 0.718; Brier score 0.155 vs. 0.155) and in the 2020 validation cohort (AUC 0.727 vs. 0.727; Brier score 0.114 vs. 0.114). These results indicate that inclusion of the interaction term did not provide incremental predictive value beyond the selected predictors (Supplementary Table S20).

## Discussion

We successfully developed and validated a predictive model using routine health examination data to identify individuals at risk of progressing to stage 4 CKM. The PREVENT equations (enhanced novel equations that predict the risk of cardiovascular events) further expanded the cardiovascular risk assessment framework by incorporating kidney function—the estimated glomerular filtration rate (eGFR) and urinary albumin-to-creatinine ratio (UACR)—into traditional models (17, 18). However, these models continue to predict future cardiovascular events rather than overall CKM progression. In recent years, some studies have begun to use CKM progression or advanced-stage occurrence as outcomes to explore the influence of environmental, metabolic, and psychological factors on CKM progression (19–21). These studies have enriched the epidemiological evidence on CKM progression. However, a common feature is that they focus on a single exposure or risk factor, primarily evaluating overall population risk using Cox or logistic regression in public databases. Current studies have not developed individually oriented multivariate prediction tools to predict progression to stage 4 CKM. Moreover, public databases typically use a regular follow-up design, whereas individual follow-up frequency in the health screening population varies widely in real-world settings. Most individuals who undergo screening cannot complete regular health checks, making it difficult to apply such models directly to routine health management. Our research, based on irregularly collected health check data in real-world settings, has achieved temporal validation of the risk prediction for CKM progression, providing new tools and evidence for the early detection of high-risk individuals.

Models based on standardized and widely available laboratory indicators are reproducible and applicable over time across different institutions. The model’s predictors closely align with the pathophysiology of cardiorenal-metabolic progression (16). Aging is closely associated with a decline in cardiac and renal function, whereas lipid imbalance and metabolic inflammation persistently indicate damage to the heart and kidneys (22). TyG and AIP reflect the dynamic balance between atherogenic lipoproteins and capture the impact of long-term metabolic disorders on the accumulation of renal damage (23–25). The CKM classification framework is relatively new and has not yet been widely adopted in all clinical settings. Future improvements or updates defined by CKM may affect the classification results and, in turn, the model’s performance. Although our model was developed based on the current CKM standards available at the time of analysis, if the classification system undergoes significant changes, it may be necessary to re-evaluate and recalibrate. However, the predictors included in our model reflect the basic cardiovascular metabolism and renal pathophysiology of CKM (such as age, blood pressure, renal function, uric acid, lipid and blood glucose indicators). These indicators remain clinically relevant as the CKM framework continues to evolve.

Recent studies have increasingly emphasized that the liver metabolic axis is also an important manifestation of metabolic dysfunction in the progression of CKM. We also analyzed the risk of CKM stage 4 progression in a cohort of patients with MASLD and diabetes. The results showed that both were independently associated with an increased risk of CKM stage 4 progression and that this risk was significantly greater when comorbidities were present. These results suggest that the abnormal liver metabolic axis may play an essential role in the deterioration of CKM. When evaluated within the elastic-net framework, neither the main effects of MASLD and diabetes nor their interaction improved predictive performance. This likely reflects the fact that the selected predictors—such as AIP, TyG, LDL-C, and other metabolic markers—already capture substantial cardiometabolic information relevant to CKM progression. In penalized regression, variables that do not provide incremental predictive information beyond the existing feature set are automatically shrunk toward zero. Accordingly, adding MASLD, diabetes, or their interaction did not materially improve model discrimination or calibration, including in temporal validation. The diagnosis of MASLD is mainly based on ultrasound results, but compared with more advanced imaging techniques or histological assessment, its sensitivity for detecting mild fatty degeneration is limited. Therefore, the prevalence of MASLD may be underestimated, and misclassification of the disease state may also occur. However, in large-scale routine health screenings, ultrasound remains the most widely available method. Conducting large-scale MR testing or histological assessment for the initial screening of MASLD is not feasible in the real world. This supports the applicability of our research results based on ultrasound diagnosis in the real world. Future studies can incorporate more sensitive imaging techniques or histological confirmation to further verify and refine the subgroup analysis related to MASLD.

The model’s predictive ability was slightly stronger in subgroups with high metabolic loads, such as diabetes and hypertension, consistent with the physiological laws of CKM progression. Metabolic disorders and elevated blood pressure are key drivers of ongoing damage to the heart-kidney metabolic axis, triggering a cascade of lipid deposition, oxidative stress, and glomerular sclerosis (26–28). Therefore, the model is more likely to identify the high-risk characteristics of this population. Subgroup analysis revealed notable calibration deviations in several clinical subgroups. Global recalibration (*α* + *β*) improved overall calibration, with most subgroups showing substantial improvement. After recalibration, CITL moved substantially toward zero and slopes became closer to 1, confirming that global recalibration effectively corrected systematic over-prediction while preserving risk ordering. In the non-hypertensive subgroup, the pre-recalibration slope was already <1 (0.932); applying *β* = 1.201 further compressed the slope to 0.776, but CITL improved from −0.797 to −0.275. Thus, absolute risk estimates still improved. If needed, subgroup-specific intercept updates can further optimize calibration without altering the slope. Interpretation of the CKM Stage 1 subgroup in the 2021 cohort requires particular caution. This subgroup included only 89 participants with three outcome events, which results in highly unstable discrimination and calibration estimates and extremely wide confidence intervals. Such instability is expected when event counts are sparse and does not necessarily indicate model misspecification. To obtain a more stable assessment, we additionally examined CKM Stage 1 participants across the pooled cohorts (2016–2021), which included 873 individuals with 87 events. In this larger sample, model discrimination remained moderate and calibration was acceptable, supporting the overall stability of model performance in early CKM populations when evaluated in adequately sized datasets. Future studies will further evaluate the model’s performance in CKM Stage 1 individuals through continued follow-up of the existing multicenter health-examination cohort and through validation in independent health-examination populations outside the Jiangbei New Area system. These additional datasets will provide larger numbers of Stage 1 progression events and allow more robust evaluation of subgroup discrimination and calibration stability in lower-risk populations.

In this health examination database, information on medication use and lifestyle factors is incomplete, introducing potential residual confounding. To address this, we combined two complementary sensitivity analyses. First, scenario-based stress testing showed preserved discrimination and stable calibration slope across all scenarios, indicating structural model robustness. Second, E-value analysis revealed that the strongest predictors (age, Hb, TyG, eGFR) required relatively strong unmeasured confounding (risk ratio ≈2) to be explained away, supporting their credibility. However, five predictors (AIP, UA, PP, LDL-C, WWI) had CI-bound E-values of 1.00, indicating their confidence intervals include the null and that even modest unmeasured confounding could render these individual associations non-significant. These complementary analyses suggest that while some individual predictors are susceptible to confounding, the model’s core risk stratification—anchored by the strongest predictors—remains robust. Future prospective studies with systematic treatment data are needed to further improve calibration and clinical applicability.

Moreover, we assessed potential nonlinearity using restricted cubic splines and performed spline-based sensitivity analyses for key predictors (Supplementary Tables S21, S22), which showed that the linear model is robust to mild deviations from linearity.

Our research also has the following limitations. Firstly, CKM staging is only evaluated during regular health check-ups. Therefore, it is impossible to directly observe the exact time at which the disease progresses to stage 4, and individuals whose condition progresses shortly after baseline can only be identified in subsequent examinations. To partially address this issue, we reported the distribution of follow-up intervals in the cohort in 2021 and conducted stratified sensitivity analyses by the tertiles of follow-up time. The results showed that the degree of distinction among layers remained largely stable (Supplementary Figure S3). Although we used a temporal validation design and required a minimum follow-up of ≥3 years for all participants, the 2021 validation cohort had a relatively shorter follow-up period than earlier cohorts. This may potentially affect the stability of discrimination and calibration estimates for longer-horizon prediction. Future studies that link health check-up data to electronic medical records and verify event dates, or apply discrete-time survival models, may better capture the dynamic characteristics of CKM development. The study population selected through our inclusion and exclusion criteria may be healthier than the average high-risk population. This could lead to an underestimation of the risk of CKM stage 4. Future research should consider including participants with fewer examination sessions or shorter follow-up periods to enhance the representativeness of the study population and further evaluate the model’s external validity. We acknowledge that all data are from Chinese medical centers and recognize that differences in ethnicity, lifestyle, access to medical care, and disease epidemiology may significantly limit the model’s applicability to non-Asian or community populations. Although some predictive factors, such as AIP, TyG, and eGFR, have been validated in other populations (29–31), the model’s applicability in different ethnic groups and medical environments still requires further verification. Future research should validate the model in independent cohorts from different countries and ethnic groups to comprehensively assess its transferability and applicability. Specifically, subject to data-access approval, we plan to conduct retrospective external validation in at least one non-Chinese population-based cohort with compatible CKM definitions and routinely available predictors, to evaluate discrimination, calibration-in-the-large, calibration slope, and clinical utility. If systematic miscalibration is observed, we will apply model recalibration strategies, including updating the intercept to correct overall risk misestimation and logistic recalibration of both the intercept and calibration slope to account for differences in baseline risk and risk gradients. This study did not conduct a prospective evaluation of whether model-based risk stratification could improve clinical decisions or patient outcomes. Therefore, the clinical impact of this model in real-world clinical scenarios still needs to be verified, and future prospective studies are required to assess its value in clinical practice. Beyond methodological validation, additional translational work will be required to operationalize the model in practice.

In conclusion, our study created an effective and scalable predictive model by using scientific outcome definitions, a time-safe validation framework, and a clinically consistent modeling strategy. This study provides a practical tool for CKM risk stratification and population health management, offering a dependable approach to complex clinical screening.

## Data Availability

The raw data supporting the conclusions of this article will be made available by the authors without undue reservation.

## Glossary

AHA: American Heart Association
AIP: Atherogenic index of plasma
AUC: area under the receiver operating characteristic curve
BMI: Body mass index
BUN: blood urea nitrogen
CKD: Chronic kidney disease
CKD-EPI: Chronic Kidney Disease Epidemiology Collaboration
CKM: cardiovascular–kidney–metabolic syndrome
CVD: Cardiovascular disease
DCA: Decision curve analysis
eGFR: estimated glomerular filtration rate
FBG: Fasting blood glucose
Hb: hemoglobin
HDL-C: high-density lipoprotein cholesterol
LDL-C: low-density lipoprotein cholesterol
LOESS: locally estimated scatterplot smoothing
MAP: mean arterial pressure
NHHR: non-HDL-C to HDL-C ratio
OBS: Oxidative balance score
OR: odds ratio
PLT: Platelet count
PP: Pulse pressure
PREVENT: Predicting the Risk of Cardiovascular Events through Enhanced Novel Equations
RC: Remnant cholesterol
SD: standard deviation
SDOH: social determinants of health
SMD: Standardized mean difference
TEST: temporal validation cohort (2021)
TG: triglyceride
TRAIN: Development (training) cohort (2016–2019)
TyG: triglyceride–glucose index
UA: Uric acid
UACR: Urinary albumin-to-creatinine ratio
VAL: Calibration (validation) cohort (2020)
WBC: white blood cell count
WWI: weight-adjusted waist index
MASLD: metabolic dysfunction-associated steatotic liver disease
