# Supplementary material for: Development and validation of a prediction model for cardiovascular–kidney–metabolic syndrome progression: a multicenter study
Source: Front Nutr. 2026 Mar 19;13:1783262. doi: 10.3389/fnut.2026.1783262 (PMC13044103; doi:10.3389/fnut.2026.1783262)
Supplement: Supplementary file 1 [file Data_Sheet_1.docx]

**Supplementary Figures and Tables**


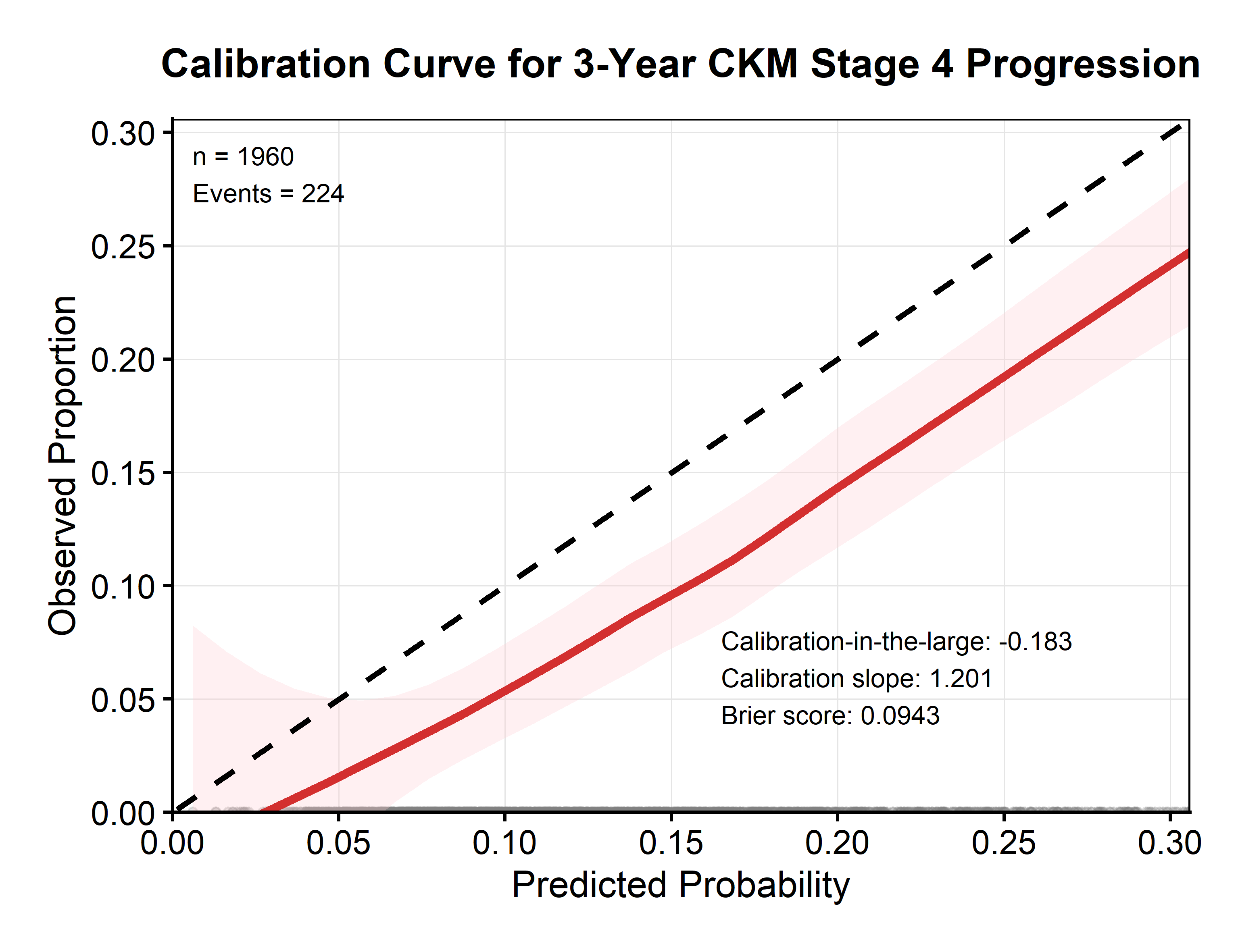


**Figure S1 The LOESS-smoothed curve for 3-year CKM stage 4 progression in the 2021 temporal validation cohort**

The solid red line represents the LOESS-smoothed observed risk, and the shaded area denotes the 95% confidence interval. The dashed diagonal line indicates perfect calibration. In the 2021 temporal validation cohort (n = 1,960; events = 224), the calibration-in-the-large was –0.183, the calibration slope 1.201, and the Brier score 0.0943.


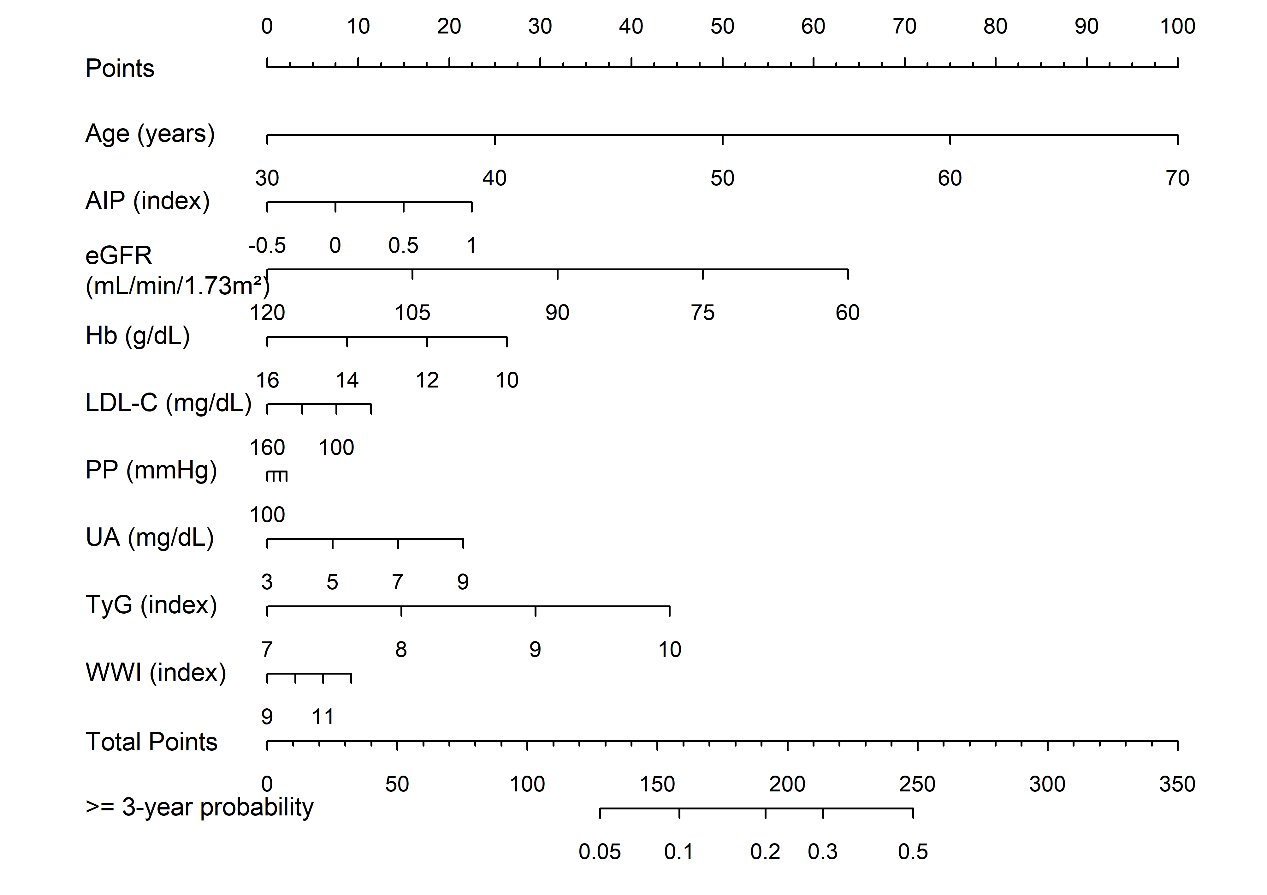


**Figure S2 Nomogram for predicting CKM stage 4 progression**

The nomogram illustrates the relative contribution of each predictor to the probability of progression to cardiovascular–kidney–metabolic (CKM) stage 4 at the next available examination (≥ 3 years after baseline). For each variable, a corresponding score can be read on the “Points” scale at the top. The total points are summed across all the variables and projected downward to estimate the individual risk of CKM stage 4 on the “Predicted probability” scale. A higher total score indicates a greater risk of CKM stage 4 progression.


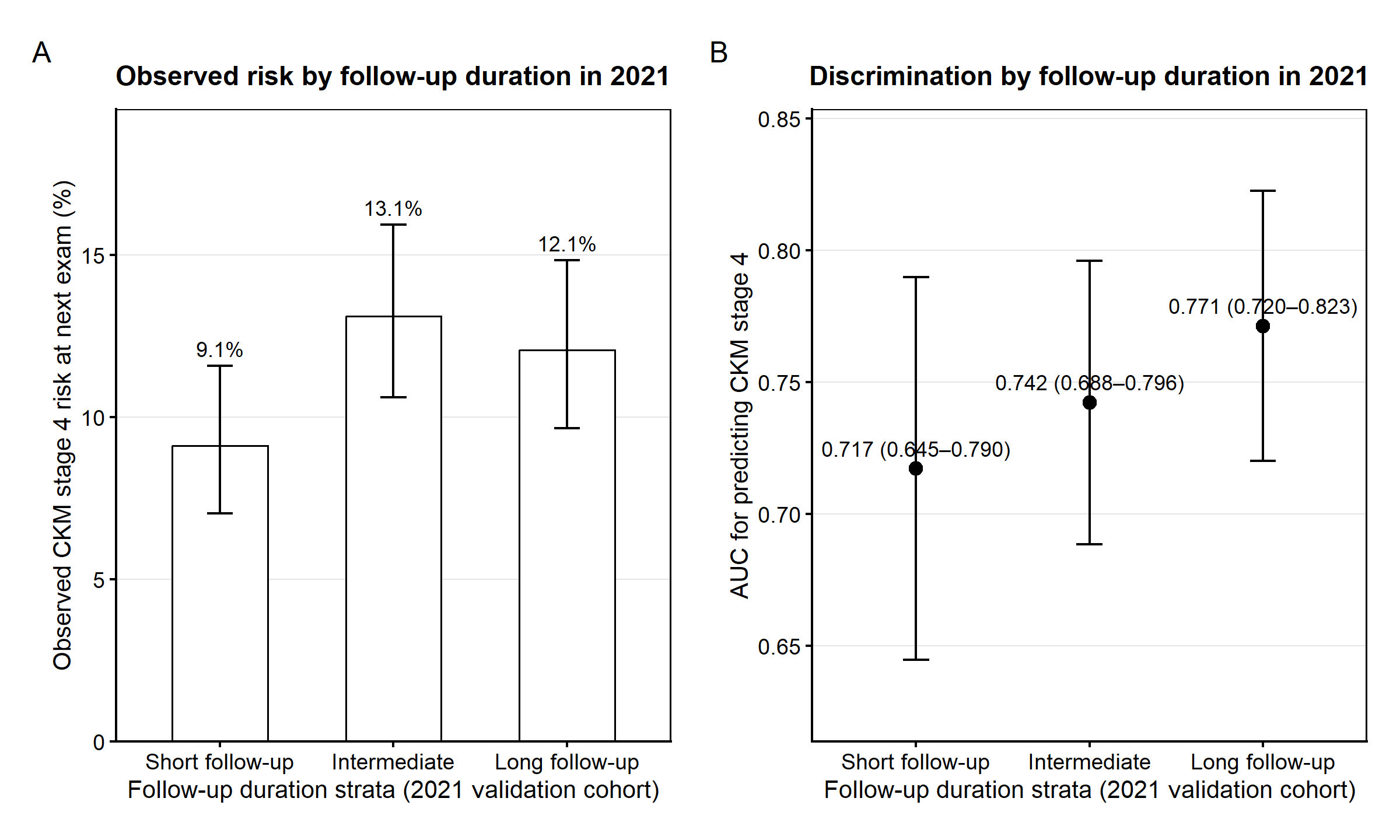


**Figure S3 Model performance across strata of follow-up duration in the 2021 cohort**

Panel A shows the observed proportions of CKM stage 4 progression at the next examination, stratified by tertiles of follow-up length (short, intermediate, and long).

Panel B presents the corresponding area under the receiver operating characteristic curve (AUC) with 95% confidence intervals in each stratum.


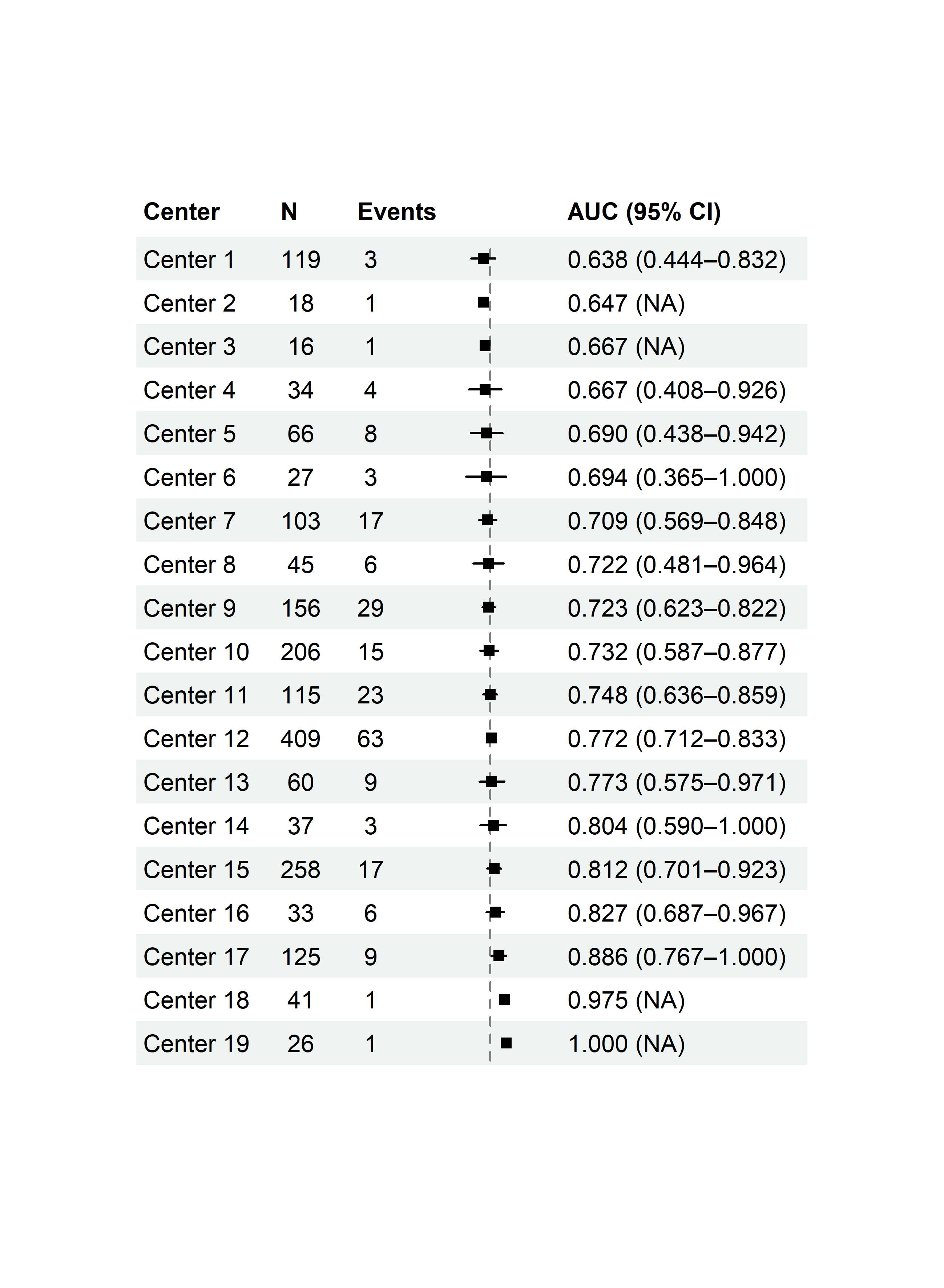


**Figure S4 Discrimination of the model across centers in the 2021 cohort**

Each point represents the area under the AUC for an individual center, with horizontal bars indicating the 95% confidence intervals. The model showed broadly consistent discrimination across these centers, with AUCs ranging approximately from 0.64 to 0.89 and largely overlapping confidence intervals. Very small centers with one or two events produced numerically high but statistically unstable estimates.


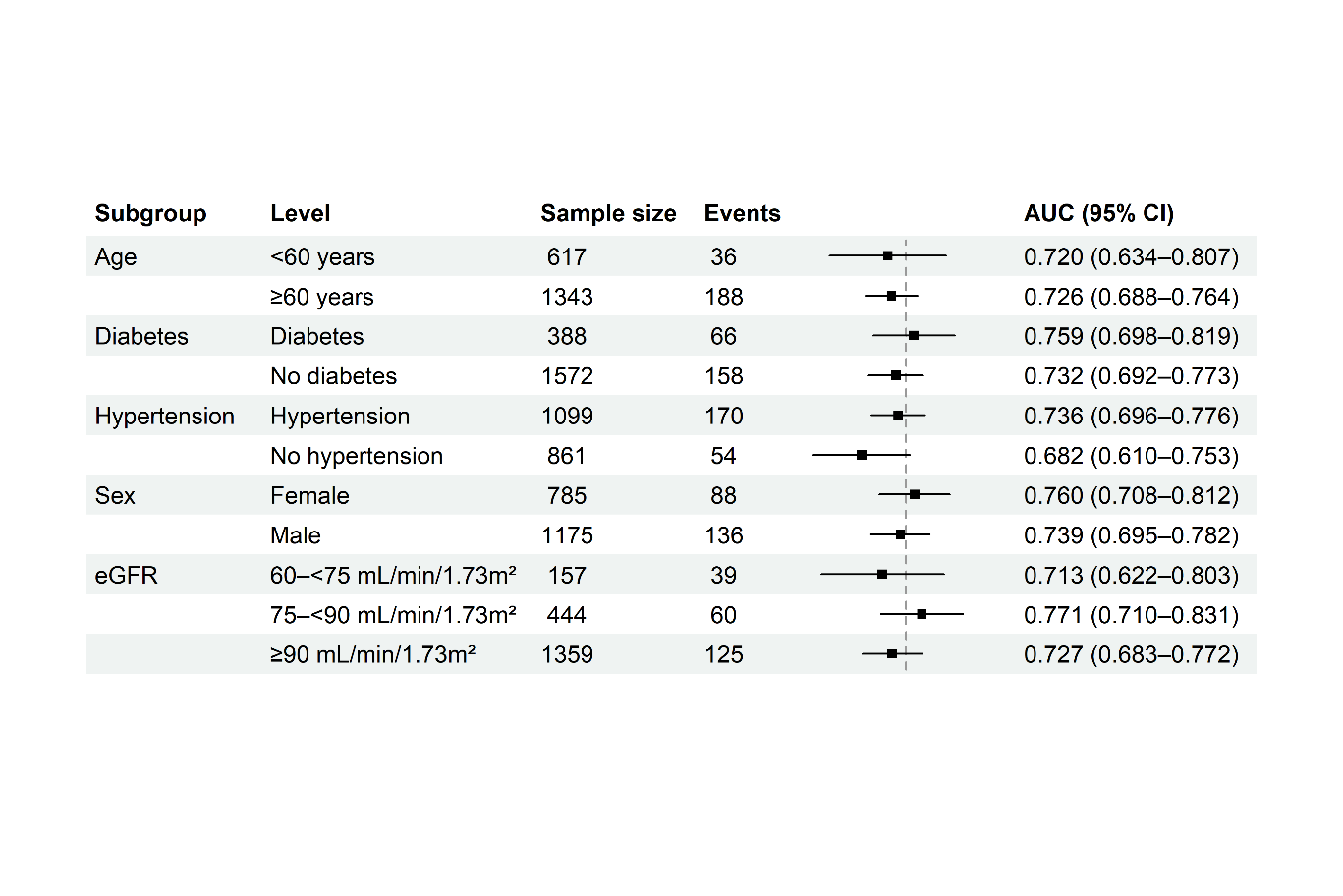


**Figure S5 Model discrimination across clinical subgroups in the 2021 cohort**

Forest plot showing the area under the receiver operating characteristic curve (AUC) and 95% confidence intervals across predefined clinical subgroups, including age (< 60 vs ≥ 60 years), sex, diabetes, hypertension, and baseline kidney function (eGFR ≥ 60 vs < 60 mL/min/1.73 m²). AUCs generally between 0.68 and 0.77 and largely overlapping confidence intervals.


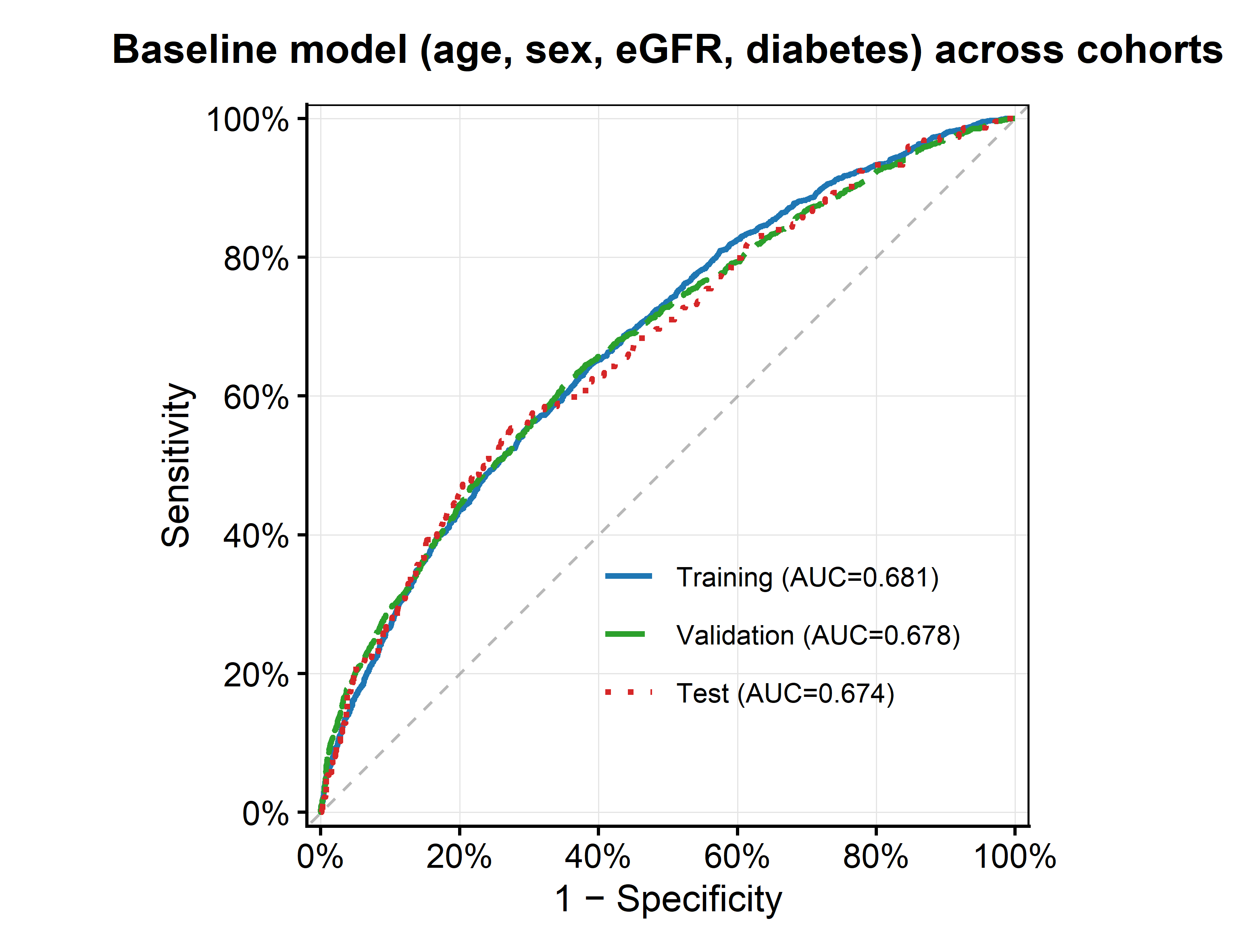


**Figure S6. Discrimination performance of a parsimonious reference model including age, sex, eGFR, and diabetes status across cohorts (2016–2021).**

The simple reference model demonstrated moderate discrimination for predicting 3-year progression to CKM stage 4 (AUC = 0.681 in the training cohort, 0.678 in the validation cohort, and 0.674 in the test cohort). The final model demonstrated significant improvements in reclassification and discrimination (continuous NRI = 0.240, 95% CI 0.106–0.377; IDI = 0.0087, 95% CI 0.0009–0.0167).

**Table S1 Quantitative assessment of potential selection bias**

| **Variable** | **Included** | | **Excluded** | **SMD** | |
| --- | --- | --- | --- | --- | --- |
| CKM progression (event rate) | 3577/21026 (17.01%) [16.51–17.53] | 5019/28750 (17.46%) [17.02–17.90] | |  |  |
|  | *P*=0.194, OR=1.03 (0.98–1.08) | | |  |  |
| Age (years) | 65.61 (7.18) | 65.23 (7.50) | | 0.051 |  |
| Smoke (yes) | 3408/21026 (16.2%) | 5089/28750 (17.7%) | | -0.04 |  |
| Alcohol (yes) | 4404/21026 (20.9%) | 6217/28750 (21.6%) | | -0.017 |  |
| BMI (kg/m²) | 25.01 (2.96) | 24.94 (3.17) | | 0.022 |  |
| Hypertension | 13085/21026 (62.2%) | 18206/28750 (63.3%) | | -0.023 |  |
| Diabetes | 5131/21026 (24.4%) | 7509/28750 (26.1%) | | -0.039 |  |
| WC (cm) | 85.63 (8.18) | 85.01 (8.79) | | 0.074 |  |
| HDL (mg/dL) | 53.41 (12.53) | 55.87 (13.12) | | -0.192 |  |
| TG (mg/dL) | 151.23 (90.62) | 138.40 (93.37) | | 0.139 |  |
| LDL (mg/dL) | 115.31 (32.32) | 114.01 (33.97) | | 0.039 |  |
| PP (mmHg) | 56.55 (13.77) | 54.77 (13.93) | | 0.128 |  |
| MAP (mmHg) | 102.58 (10.54) | 102.53 (10.84) | | 0.005 |  |
| eGFR  (mL/min/1.73 m²) | 91.95 (11.55) | 92.69 (12.92) | | -0.06 |  |
| FBG (mmol/L) | 5.95 (1.55) | 5.98 (1.79) | | -0.019 |  |

Continuous variables are presented as mean (SD), and categorical variables as N (%). Standardized mean differences (SMDs) quantify baseline imbalance. Outcome event rates are presented as events/N (%) with exact 95% confidence intervals. Standardized mean differences (SMDs) were used to quantify baseline imbalance between groups; |SMD| < 0.10 indicates negligible imbalance, 0.10–0.20 mild imbalance, and > 0.20 moderate imbalance. Abbreviations: WC, waist circumference; HDL, high-density lipoprotein cholesterol; TG, triglycerides; LDL, low-density lipoprotein cholesterol; PP, pulse pressure; MAP, mean arterial pressure; eGFR, estimated glomerular filtration rate; FBG, fasting blood glucose.

**Table S2. Extreme-case sensitivity analysis for missing predictors**

| **Scenario** | **AUC** | **CITL** | **Slope** |
| --- | --- | --- | --- |
| Complete-case | 0.7039 | -0.5066 | 0.9769 |
| 5th percentile fill | 0.7002 | -0.4718 | 1.0097 |
| 95th percentile fill | 0.7002 | -0.4718 | 1.0097 |

An extreme-case sensitivity analysis was conducted to assess the robustness of the complete-case approach to missing predictor variables. In the development cohort (2016–2019), missing values of key predictors were imputed using the 5th or 95th percentile of the observed distributions (representing conservative low-risk and high-risk scenarios, respectively). Under each scenario, a simplified logistic regression model was refitted using the same set of predictors as the final model. Model performance (AUC, calibration-in-the-large [CITL], and calibration slope) was evaluated in the fixed 2021 complete-case temporal validation cohort.

**Table S3. Cutoffs for Extreme Values (0.2th and 99.8th percentiles)**

| **Variable** | **Lower (0.2th)** | **Upper (99.8th)** | **Outside Range** |
| --- | --- | --- | --- |
| TG (mg/dL) | 12.18 | 765.89 | 160 |
| TC (mg/dL) | 97.06 | 345.81 | 155 |
| HDL-C (mg/dL) | 26.30 | 113.30 | 151 |
| LDL-C (mg/dL) | 32.87 | 224.67 | 155 |
| FBG (mmol/L) | 3.62 | 16.90 | 159 |
| eGFR (mL/min/1.73 m²) | 60.31 | 122.72 | 158 |
| Scr (mg/dL) | 0.37 | 1.30 | 129 |
| BUN (mmol/L) | 3.50 | 10.79 | 80 |
| WBC (×10⁹/L) | 2.90 | 13.08 | 160 |
| ALT/AST | 5 | 122 | 156 |
| AST | 5 | 95.72 | 152 |
| Total bilirubin (µmol/L) | 3.6 | 45.85 | 148 |
| BMI (kg/m²) | 17.20 | 36.83 | 160 |
| WC (cm) | 62.58 | 115 | 142 |
| MAP (mmHg) | 73.33 | 140 | 150 |
| PP (mmHg) | 23 | 110 | 156 |

Extreme values were defined using the 0.2th and 99.8th percentiles calculated in the development cohort. Participants with any value outside these thresholds were excluded. This procedure was pre-specified to mitigate the influence of implausible outliers.

**Table S4. Candidate predictors and feature-reduction pipeline**

| **Domain** | **Candidate predictors** | **Retained in final model** |
| --- | --- | --- |
| Baseline/demographics & comorbidities | Age, Sex, Hypertension, Diabetes, Fatty liver | Age |
| Anthropometry/adiposity | BMI, WHtR, RFM, VAI, BRI, WWI, LAP | WWI |
| Blood pressure/hemodynamic load | MAP, PP | PP |
| Renal–uric acid metabolism | eGFR, BUN, UA, UHR, UA/Cr | eGFR, UA |
| Lipid fractions | HDL-C, LDL-C, non-HDL-C, RC | LDL-C |
| Lipid ratios | AIP, TG/HDL, Castelli I, Castelli II, NHHR | AIP |
| Glycaemic / insulin resistance | FBG, TyG | TyG |
| Inflammatory/haematological | WBC, Hb, PLT, PHR | Hb |

Candidate predictors were grouped into clinically coherent domains. Within each domain, correlation-based clustering was used to reduce redundancy, and representative features were selected to enter penalized modeling.

**Table S5 Final model coefficients and standardization parameters**

| **Variable** | **Transformation** | **Mean** | **SD** | **β** | **OR**  **per 1 SD** | |
| --- | --- | --- | --- | --- | --- | --- |
| Intercept | Intercept | NA | NA | -1.9392 | 0.1438 |  |
| Age (years) | z-score | 66.49 | 7.36 | 0.4293 | 1.54 |  |
| AIP (index) |  | 0.418 | 0.258 | 0.1574 | 1.17 |  |
| eGFR (mL/min/1.73 m²) |  | 91.23 | 12.01 | -0.3214 | 0.73 |  |
| Hb (g/L) |  | 139.88 | 14.11 | -0.1434 | 0.87 |  |
| LDL-C  (mg/dL) |  | 111.32 | 34.31 | -0.0619 | 0.94 |  |
| PP (mmHg) |  | 57.50 | 13.78 | -0.0208 | 0.98 |  |
| UA (mg/dL) |  | 5.14 | 1.37 | 0.0555 | 1.06 |  |
| TyG (index) |  | 8.86 | 0.54 | 0.0554 | 1.06 |  |
| WWI (cm/√kg) |  | 10.57 | 0.75 | 0.0270 | 1.03 |  |

Continuous predictors were standardized using z-scores based on the mean and standard deviation of the training cohort: z = (x − mean_train) / SD_train. The β coefficients correspond to the log-odds per 1 SD increase in each standardized predictor. The individual linear predictor (LP) is calculated as: LP=β_0+∑_(i=1)^k▒β_i z_i. The predicted 3-year probability of CKM stage 4 progression is obtained by: P=1/(1+exp⁡(-LP)). Definitions of derived indices: AIP = log10[TG (mg/dL) / HDL-C (mg/dL)]; TyG = ln[TG (mg/dL) × FBG (mg/dL) / 2]; WWI = WC (cm) / √weight (kg). AIP and TyG are unitless indices; WWI is expressed as cm/√kg.

Abbreviations: AIP, atherogenic index of plasma; eGFR, estimated glomerular filtration rate; Hb, hemoglobin; LDL-C, low-density lipoprotein cholesterol; PP, pulse pressure; UA, uric acid; TyG, triglyceride-glucose index; WWI, weight-adjusted waist index.

**Table S6 Composition of the analytic cohort by type of center (2016–2024)**

| **Center type** | **Number of centers** | **Participants (n)** | **Events (n)** | **Event rate (%)** |
| --- | --- | --- | --- | --- |
| Community health service center | 44 | 14,770 | 2,722 | 18.4 |
| Hospital | 51 | 4,068 | 476 | 11.7 |
| Outpatient department | 6 | 1,653 | 308 | 18.6 |
| Township hospital / health station | 4 | 298 | 42 | 14.1 |
| Clinic | 1 | 143 | 18 | 12.6 |
| Other | 6 | 57 | 5 | 8.8 |
| Health examination center | 8 | 37 | 6 | 16.2 |
| Total | 120 | 21,026 | 3,577 | 17.0 |

The table summarizes the distribution of participating centers, sample sizes, and CKM stage 4 events across different types of institutions in the 2016–2024 analytic cohort. A total of 120 independent centers were included, comprising hospitals, community health service centers, outpatient departments, township hospitals or health stations, clinics, and health examination centers.

**Table S7 Composition of CKM stage 4 events in the 2016–2021 cohort**

| **Category** | **Events (n)** | **Proportion, %** |
| --- | --- | --- |
| CVD only | 2640 | 74.3 |
| CVD + eGFR<60 | 914 | 25.7 |

The table shows the composition of CKM stage 4 events, based on whether CVD was present and whether renal function was impaired at the most recent available examination.

**Table S8** **Model performance in cohorts**

| **Dataset** | **Sample size**  **(n)** | **Events**  **(n)** | **AUC (95% CI)** | **Calibration-in-the-large** | **Calibration slope** | **Brier score** |
| --- | --- | --- | --- | --- | --- | --- |
| TRAIN | 7,831 | 1,767 | 0.718 (0.704–0.730) |  |  |  |
| VAL | 11,235 | 1,586 | 0.727 (0.714–0.740) |  |  |  |
| TEST | 1,960 | 224 | 0.747 (0.711–0.777) | -0.183 | 1.201 | 0.0943 |

The table summarizes the discrimination and calibration performance of the final elastic-net model across the time-split datasets. The model showed consistent discrimination across all temporal cohorts, with AUCs of 0.718 (95% CI, 0.704–0.730) in the 2016–2019 development cohort, 0.727 (95% CI, 0.714–0.740) in the 2020 calibration cohort, and 0.747 (95% CI, 0.711–0.777) in the 2021 temporal validation cohort. CI, confidence interval; TRAIN, development (training) cohort; VAL, calibration cohort; TEST, temporal validation cohort.

**Table S9 Observed event rates across predicted-risk quartiles in the 2021 test cohort.**

| **Risk quartile** | **N** | **Events** | **Event rate (%)** | **95% CI** |  |
| --- | --- | --- | --- | --- | --- |
| Q1 | 490 | 9 | 1.8 | 0.8–3.5 | |
| Q2 | 490 | 37 | 7.6 | 5.4–10.3 | |
| Q3 | 490 | 56 | 11.4 | 8.7–14.6 | |
| Q4 | 490 | 122 | 24.9 | 21.1–29.0 | |

Observed event rates and 95% confidence intervals (CIs) were calculated using binomial exact methods. Quartiles were defined based on predicted risks from the final model in the 2021 external test cohort.

**Table S10. Performance across follow-up duration tertiles in the 2021 cohort**

| **Follow-up** | **N** | **Events** | **AUC**  **(95% CI)** | **Calibration intercept (95% CI)** | **Calibration slope (95% CI)** |
| --- | --- | --- | --- | --- | --- |
| Short | 644 | 60 | 0.719  (0.647–0.791) | −0.545  (−1.115–−0.016) | 1.096  (0.750–1.437) |
| Intermediate | 662 | 85 | 0.746  (0.692–0.799) | −0.044  (−0.612–0.510) | 1.200  (0.844–1.552) |
| Long | 654 | 79 | 0.766  (0.714–0.818) | 0.023  (−0.522–0.627) | 1.321  (0.984–1.715) |

AUC indicates discrimination. Calibration intercept (CITL) reflects systematic over- or under-prediction (ideal = 0), and calibration slope reflects agreement between predicted and observed risk gradients (ideal = 1). Calibration metrics were obtained using logistic calibration with bootstrap 95% confidence intervals.

**Table S11 Sensitivity analysis with baseline eGFR ≥ 65 in the 2021 cohort**

| **Subset** | **Sample size**  **(n)** | **Events**  **(n)** | **AUC** | **95% CI**  **(lower)** | **95% CI**  **(upper)** |
| --- | --- | --- | --- | --- | --- |
| eGFR>=65 at baseline | 1,920 | 209 | 0.7419987 | 0.7071944 | 0.776803 |

The table presents the model’s discrimination performance in a sensitivity analysis restricted to participants with preserved baseline kidney function (eGFR ≥ 65 mL/min/1.73 m²) within the 2021 temporal validation cohort. After excluding participants with reduced baseline eGFR, the model retained an AUC of 0.742 (95% CI 0.707–0.777.

**Table S12 Center discrimination performance in the 2021 cohort**

| **Center** | **Center name** | **Sample size (n)** | **Events (n)** | **AUC (95% CI)** |
| --- | --- | --- | --- | --- |
| Center 1 | Puji Hospital | 119 | 3 | 0.638 (0.444–0.832) |
| Center 2 | Qiao Yin Road Community Health Service Station | 18 | 1 | 0.647 (NA) |
| Center 3 | Zhongda Hospital Affiliated to Southeast University | 16 | 1 | 0.667 (NA) |
| Center 4 | Changhai Health Clinic | 34 | 4 | 0.667 (0.408–0.926) |
| Center 5 | Dahua Clinic | 66 | 8 | 0.690 (0.438–0.942) |
| Center 6 | The Fourth Affiliated Hospital of Nanjing Medical University | 27 | 3 | 0.694 (0.365–1.000) |
| Center 7 | Guankang Road Community Health Service Center of Jiangbei New District, Nanjing City | 103 | 17 | 0.709 (0.569–0.848) |
| Center 8 | Xiangyi Zijun Clinic | 45 | 6 | 0.722 (0.481–0.964) |
| Center 9 | Dachang Community Health Service Center of Jiangbei New District, Nanjing City | 156 | 29 | 0.723 (0.623–0.822) |
| Center 10 | Nanjing Yangzi Hospital | 206 | 15 | 0.732 (0.587–0.877) |
| Center 11 | Taishan Sub-district Community Health Service Center of Jiangbei New District, Nanjing City | 115 | 23 | 0.748 (0.636–0.859) |
| Center 12 | Jiangning New District, Top Mountain Sub-district, Community Health Service Center, Nanjing City | 409 | 63 | 0.772 (0.712–0.833) |
| Center 13 | Nanjing Zhikang Clinic | 60 | 9 | 0.773 (0.575–0.971) |
| Center 14 | Xiejiadian Community Health Service Center of Jiangbei New District, Nanjing City | 37 | 3 | 0.804 (0.590–1.000) |
| Center 15 | Pan Cheng Sub-district Community Health Service Center of Jiangbei New District, Nanjing City | 258 | 17 | 0.812 (0.701–0.923) |
| Center 16 | Nanjing Jiangbei People's Hospital | 33 | 6 | 0.827 (0.687–0.967) |
| Center 17 | Jiang River Street Community Health Service Center | 125 | 9 | 0.886 (0.767–1.000) |
| Center 18 | Tianrun Traditional Chinese Medicine Clinic | 41 | 1 | 0.975 (NA) |
| Center 19 | Kangmin Integrated Traditional Chinese and Western Medicine Clinic | 26 | 1 | 1.000 (NA) |

Centers in the 2021 validation cohort, center-specific AUCs ranged from 0.64 to 0.89 with largely overlapping 95% CIs. In several very small centers (≤2 events), AUC estimates were numerically high (up to 1.00) but associated with extremely wide or non-estimable confidence intervals.

**Table S13 Calibration of the final model in the 2021 temporal validation cohort**

| **Center** | **Sample** | **Events** | **CITL (95% CI)** | **Slope (95% CI)** | **E/O (95% CI)** |
| --- | --- | --- | --- | --- | --- |
| Center 1 | 119 | 3 | -2.08 (-24.88, -1.23) | 0.44 (-0.29, 1.53) | 0.16 (0.00, 0.35) |
| Center 2 | 18 | 1 | -1.45 (-24.26, -0.04) | 0.93 (-0.00, 2.42) | 0.29 (0.00, 0.97) |
| Center 3 | 16 | 1 | -1.44 (-24.42, -0.01) | 0.89 (-0.00, 4.16) | 0.30 (0.00, 0.99) |
| Center 4 | 34 | 4 | -0.37 (-1.99, 0.52) | 0.81 (-0.42, 3.72) | 0.74 (0.18, 1.49) |
| Center 5 | 66 | 8 | -0.31 (-1.24, 0.35) | 0.84 (-0.78, 3.01) | 0.78 (0.35, 1.31) |
| Center 6 | 27 | 3 | -0.67 (-23.96, 0.27) | 1.32 (-0.80, 69.88) | 0.59 (0.00, 1.21) |
| Center 7 | 103 | 17 | 0.08 (-0.53, 0.54) | 1.06 (0.30, 2.32) | 1.06 (0.65, 1.51) |
| Center 8 | 45 | 6 | -0.41 (-1.68, 0.35) | 1.07 (-0.16, 5.20) | 0.73 (0.24, 1.28) |
| Center 9 | 156 | 29 | 0.30 (-0.18, 0.67) | 1.15 (0.56, 2.04) | 1.26 (0.86, 1.65) |
| Center 10 | 206 | 15 | -1.21 (-1.82, -0.72) | 1.04 (0.45, 1.98) | 0.38 (0.22, 0.57) |
| Center 11 | 115 | 23 | 0.17 (-0.40, 0.61) | 1.00 (0.44, 1.90) | 1.13 (0.74, 1.52) |
| Center 12 | 409 | 63 | -0.02 (-0.32, 0.22) | 1.57 (1.19, 2.02) | 0.98 (0.77, 1.18) |
| Center 13 | 60 | 9 | -0.11 (-0.97, 0.47) | 1.42 (0.27, 3.39) | 0.92 (0.44, 1.40) |
| Center 14 | 37 | 3 | -0.89 (-24.73, -0.01) | 1.60 (0.00, 9.69) | 0.48 (0.00, 1.00) |
| Center 15 | 258 | 17 | -1.13 (-1.72, -0.71) | 1.57 (1.00, 2.38) | 0.40 (0.23, 0.57) |
| Center 16 | 33 | 6 | -0.34 (-1.50, 0.47) | 1.43 (0.60, 5.66) | 0.79 (0.30, 1.37) |
| Center 17 | 125 | 9 | -0.76 (-1.62, -0.27) | 2.49 (1.37, 5.43) | 0.53 (0.24, 0.81) |
| Center 18 | 41 | 1 | -1.88 (-24.76, -0.73) | 7.64 (-0.00, 253.21) | 0.18 (0.00, 0.53) |
| Center 19 | 26 | 1 | -1.22 (-23.56, -0.02) | 202.77 (-0.00, 213.28) | 0.33 (0.00, 0.99) |

Calibration intercept (CITL), calibration slope, and expected/observed (E/O) ratio with 95% confidence intervals were estimated via bootstrap resampling (1,000 replicates). Ideal calibration is indicated by CITL = 0, slope = 1, and E/O = 1. Centers with very few events (≤3 events) exhibit extremely wide or non‑estimable confidence intervals, reflecting expected instability under sparse events. Among centers with ≥15 events, CITL and slope confidence intervals generally contain the ideal values, and E/O ratios are close to 1, indicating good calibration in centers with reliable sample sizes.

**Table S14 Discrimination and calibration across clinically relevant subgroups in the 2021 cohort**

| **Subgroup** | **Level** | **Sample** | **Events** | **AUC (95% CI)** | **CITL (95% CI)** | **Slope (95% CI)** |
| --- | --- | --- | --- | --- | --- | --- |
| Age | <60 years | 617 | 36 | 0.720  (0.634–0.807) | -0.704  (-1.119–-0.398) | 1.400 (0.819–2.086) |
|  | ≥60 years | 1343 | 188 | 0.726  (0.688–0.764) | -0.397  (-0.553–-0.250) | 1.129 (0.916–1.386) |
| Diabetes | Yes | 388 | 66 | 0.759  (0.698–0.819) | -0.216  (-0.483–0.041) | 1.184 (0.832–1.623) |
|  | No | 1572 | 158 | 0.732  (0.692–0.773) | -0.540  (-0.712–-0.388) | 1.169 (0.949–1.417) |
| Hypertension | Yes | 1099 | 170 | 0.736  (0.696–0.776) | -0.308  (-0.483–-0.154) | 1.188 (0.963–1.467) |
|  | No | 861 | 54 | 0.682  (0.610–0.753) | -0.797  (-1.104–-0.553) | 0.932 (0.543–1.344) |
| Sex | Female | 785 | 88 | 0.760  (0.708–0.812) | -0.496  (-0.718–-0.288) | 1.348 (1.025–1.722) |
|  | Male | 1175 | 136 | 0.739  (0.695–0.782) | -0.434  (-0.626–-0.256) | 1.119 (0.880–1.390) |
| eGFR  (mL/min/1.73m²) | 60–<75 | 157 | 39 | 0.713  (0.622–0.803) | -0.431  (-0.830–-0.074) | 1.097 (0.570–1.759) |
|  | 75–<90 | 444 | 60 | 0.771  (0.710–0.831) | -0.631  (-0.935–-0.377) | 1.568 (1.130–2.156) |
|  | ≥90 | 1359 | 125 | 0.727  (0.683–0.772) | -0.382  (-0.589–-0.217) | 1.369 (1.065–1.662) |
| MASLD | Yes | 850 | 135 | 0.712 (0.664–0.761) | -0.301 (-0.506–-0.118) | 1.090 (0.814–1.391) |
|  | No | 1110 | 89 | 0.751 (0.701–0.802) | -0.647 (-0.862–-0.449) | 1.231 (0.947–1.548) |
| Baseline CKM stage | Stage 1 | 89 | 3 | 0.764 (0.488–1.000) | -1.253 (-2.430–-0.424) | 2.029 (0.023–1174.574)  Unstable (few events) |
|  | Stage 2 | 1871 | 221 | 0.742 (0.708–0.776) | -0.440 (-0.595–-0.291) | 1.177 (0.982–1.377) |

AUC indicates discrimination. Calibration intercept (calibration-in-the-large) reflects systematic over- or underestimation of absolute risk (ideal value = 0), and calibration slope reflects agreement between predicted and observed risk gradients (ideal value = 1). Calibration metrics were estimated using logistic calibration with bootstrap 95% confidence intervals in the 2021 temporal validation cohort.

**Table S15 Subgroup calibration after global α+β recalibration (2021 cohort)**

| **Subgroup** | **Level** | **Sample** | **Events** | **AUC (95% CI)** | **CITL (after)** | **Slope (after)** |
| --- | --- | --- | --- | --- | --- | --- |
| Age | <60 years | 617 | 36 | 0.720  (0.634–0.807) | -0.140 (-0.514–0.169) | 1.165 (0.672–1.763) |
|  | ≥60 years | 1343 | 188 | 0.726  (0.688–0.764) | 0.034 (-0.135–0.195) | 0.940 (0.757–1.132) |
| Diabetes | Yes | 388 | 66 | 0.759  (0.698–0.819) | -0.039 (-0.270–0.181) | 1.122 (0.864–1.426) |
|  | No | 1572 | 158 | 0.732  (0.692–0.773) | 0.026 (-0.176–0.198) | 0.932 (0.735–1.151) |
| Hypertension | Yes | 1099 | 170 | 0.736  (0.696–0.776) | -0.068 (-0.246–0.096) | 0.973 (0.786–1.184) |
|  | No | 861 | 54 | 0.682  (0.610–0.753) | 0.202 (-0.103–0.482) | 0.986 (0.698–1.334) |
| Sex | Female | 785 | 88 | 0.760  (0.708–0.812) | -0.275 (-0.581–0.003) | 0.776 (0.461–1.110) |
|  | Male | 1175 | 136 | 0.739  (0.695–0.782) | 0.119 (-0.063–0.294) | 0.989 (0.798–1.198) |
| eGFR  (mL/min/1.73m²) | 60–<75 | 157 | 39 | 0.713  (0.622–0.803) | -0.132 (-0.546–0.231) | 0.913 (0.469–1.489) |
|  | 75–<90 | 444 | 60 | 0.771  (0.710–0.831) | -0.233 (-0.526–0.023) | 1.305 (0.919–1.797) |
|  | ≥90 | 1359 | 125 | 0.727  (0.683–0.772) | 0.151 (-0.046–0.330) | 1.140 (0.881–1.411) |
| MASLD | Yes | 850 | 135 | 0.712 (0.664–0.761) | -0.151 (-0.393–0.064) | 1.025 (0.788–1.282) |
|  | No | 1110 | 89 | 0.751 (0.701–0.802) | 0.125 (-0.076–0.315) | 0.908 (0.673–1.173) |
| Baseline CKM stage | Stage 1 | 89 | 3 | 0.764 (0.488–1.000) | -0.684 (-1.863–0.209) | 1.690 (0.042–965.686) |
|  | Stage 2 | 1871 | 221 | 0.742 (0.708–0.776) | 0.015 (-0.132–0.161) | 0.980 (0.815–1.158) |

Global recalibration: logit(y) = α + β·logit(p), with α = −0.183, β = 1.201 estimated in the full 2021 cohort. AUC remains unchanged after recalibration because the transformation is monotonic and does not alter risk ranking. CITL and slope confidence intervals were obtained by bootstrap (2,000 replicates).

**Table S16. Model performance in CKM Stage-1 participants across study cohorts**

| **Cohort** | **N** | **Events** | **AUC (95% CI)** | **CITL (95% CI)** | **Slope (95% CI)** | |
| --- | --- | --- | --- | --- | --- | --- |
| 2021 cohort | 89 | 3 | 0.764  (0.477–1.000) | 0.601  (−26.566–907.196) | 2.029  (~0–1163.619) | |
| 2016–2021 cohort | 873 | 87 | 0.676  (0.609–0.731) | −0.244  (−0.920–0.435) | 1.061  (0.707–1.459) |  |

The pooled cohort combines CKM Stage-1 participants from the development and temporal validation cohorts. Because the pooled dataset includes individuals used in model development, this analysis is presented as a descriptive stability assessment rather than an independent external validation. Estimates from the 2021 cohort should be interpreted cautiously due to the extremely small number of events (n=3).

**Table S17. Scenario-based Stress Testing of Model Performance (TEST 2021)**

| **Scenario** | **AUC**  **(95% CI)** | **Brier score**  **(95% CI)** | **CITL**  **(95% CI)** | **Slope**  **(95% CI)** |
| --- | --- | --- | --- | --- |
| Baseline | 0.747  (0.713, 0.779) | 0.0943  (0.0861, 0.1027) | -0.183  (-0.493, 0.125) | 1.20  (1.00, 1.41) |
| eGFR  (mL/min/1.73 m²) | 0.741  (0.703, 0.773) | 0.0951  (0.0867, 0.1033) | -0.357  (-0.680, -0.084) | 1.10  (0.91, 1.28) |
| Age (years) | 0.745  (0.714, 0.778) | 0.0955  (0.0874, 0.1036) | -0.408  (-0.670, -0.127) | 1.07  (0.91, 1.25) |
| Hb (g/L) | 0.748  (0.713, 0.778) | 0.0942  (0.0860, 0.1027) | -0.188  (-0.501, 0.095) | 1.20  (1.01, 1.39) |
| UA (mg/dL) | 0.746  (0.711, 0.778) | 0.0944  (0.0864, 0.1032) | -0.197  (-0.513, 0.122) | 1.19  (0.99, 1.40) |
| AIP (index) | 0.746  (0.713, 0.781) | 0.0945  (0.0865, 0.1033) | -0.227  (-0.535, 0.099) | 1.18  (0.98, 1.39) |
| TyG (index) | 0.746  (0.714, 0.780) | 0.0944  (0.0862, 0.1027) | -0.197  (-0.488, 0.111) | 1.19  (1.00, 1.41) |
| PP (mmHg) | 0.747  (0.714, 0.781) | 0.0943  (0.0853, 0.1023) | -0.178  (-0.511, 0.151) | 1.20  (1.01, 1.42) |
| LDL-C  (mg/dL) | 0.747  (0.710, 0.780) | 0.0943  (0.0854, 0.1030) | -0.187  (-0.508, 0.127) | 1.20  (1.00, 1.41) |
| WWI (cm/√kg) | 0.746  (0.713, 0.780) | 0.0943  (0.0863, 0.1021) | -0.186  (-0.479, 0.121) | 1.20  (1.02, 1.40) |
| Combined scenario |  |  |  |  |
| TyG+AIP ↑, eGFR ↓, PP SD×1.25 | 0.747  (0.713, 0.779) | 0.0965  (0.0885, 0.1050) | -0.339  (-0.631, -0.045) | 1.20  (1.00, 1.41) |

Stable AUC and calibration slope indicate preserved discrimination and relative calibration, whereas systematic shifts in CITL under adverse scenarios reflect potential risk underestimation and the need for recalibration in worse metabolic profiles.

**Table S18 E-values for Predictors (TEST 2021)**

| **Scenario** | **OR** | **CI** | **E-value** | **CI-bound E-value** |
| --- | --- | --- | --- | --- |
| eGFR  (mL/min/1.73 m²) | 0.76 | 0.758 (0.641–0.897) | 1.97 | 1.47 |
| Age (years) | 1.50 | 1.498 (1.265–1.774) | 2.36 | 1.84 |
| Hb (g/L) | 0.70 | 0.703 (0.599–0.824) | 2.20 | 1.72 |
| UA (mg/dL) | 1.06 | 1.061 (0.901–1.250) | 1.32 | 1.00 |
| AIP (index) | 1.07 | 1.068 (0.778–1.466) | 1.34 | 1.00 |
| TyG (index) | 1.35 | 1.347 (0.977–1.857) | 2.03 | 1.00 |
| PP (mmHg) | 0.97 | 0.966 (0.833–1.120) | 1.23 | 1.00 |
| LDL-C  (mg/dL) | 0.81 | 0.810 (0.690–0.951) | 1.77 | 1.29 |
| WWI (cm/√kg) | 1.02 | 1.019 (0.878–1.182) | 1.16 | 1.00 |

E-values were calculated based on adjusted odds ratios (per 1 SD increase) in the 2021 temporal validation cohort. The E-value represents the minimum strength of association (on the risk ratio scale) that an unmeasured confounder would need to have with both the predictor and CKM progression, conditional on measured covariates, to fully explain away the observed association. For confidence intervals including the null, the CI-bound E-value equals 1.00.

**Table S19 Association of MASLD and diabetes with CKM stage 4 progression.**

| **Exposure** | **N** | **Events** | **OR (95% CI)** | ***P* value** |
| --- | --- | --- | --- | --- |
| MASLD (vs no MASLD) | 21,026 | 3,577 | 1.62 (1.51–1.74) | <0.001 |
| Adjusted MASLD (vs no MASLD) |  |  | 1.61 (1.48–1.75) | <0.001 |
| MASLD only  (vs no diabetes and no MASLD) |  |  | 1.75 (1.60–1.92) | <0.001 |
| Diabetes only  (vs no diabetes and no MASLD) |  |  | 1.72 (1.53–1.93) | <0.001 |
| Diabetes & MASLD  (vs no diabetes and no MASLD) |  |  | 2.13 (1.89–2.40) | <0.001 |
| MASLD × diabetes (multiplicative interaction) |  |  | 0.71 (0.60–0.84) | <0.001 |

Odds ratios (ORs) and 95% confidence intervals (CIs) were estimated using logistic regression. The interaction term represents multiplicative effect modification between MASLD and diabetes. Adjusted model included age, sex, baseline eGFR, hypertension, and body mass index (BMI) as covariates.

**Table S20 Comparison of elastic-net models with and without the MASLD× Diabetes interaction**

| **Cohort** | **n** | **Events** | **Model** | **AUC** | **Brier** | **CITL** | **Slope** |
| --- | --- | --- | --- | --- | --- | --- | --- |
| TEST (2021) | 1960 | 224 | No interaction | 0.747 | 0.0943 | -0.183 | 1.20 |
|  |  |  | With MASLD×T2D | 0.746 | 0.0943 | -0.182 | 1.20 |
| TRAIN (2016–2019) | 7831 | 1767 | No interaction | 0.718 | 0.155 | 0.104 | 1.10 |
|  |  |  | With MASLD×T2D | 0.718 | 0.155 | 0.105 | 1.10 |
| VAL (2020) | 11235 | 1586 | No interaction | 0.727 | 0.114 | -0.286 | 1.18 |
|  |  |  | With MASLD×T2D | 0.727 | 0.114 | -0.286 | 1.18 |

The interaction term (MASLD×diabetes) was forced into the elastic-net model. Inclusion of the interaction did not materially improve discrimination, overall prediction error, or calibration across development, internal validation, or temporal validation cohorts.

**Table S21 Univariate nonlinearity test using restricted cubic splines**

| **Variable** | **Chi-square (Nonlinear)** | **df** | **P for nonlinearity** |
| --- | --- | --- | --- |
| eGFR (mL/min/1.73 m²) | 25.80 | 2 | 2.50E-06 |
| Age (years) | 6.32 | 2 | 0.0424 |
| Hb (g/L) | 1.51 | 2 | 0.469 |
| UA (mg/dL) | 34.29 | 2 | 3.57E-08 |
| AIP (index) | 153.91 | 2 | 3.79E-34 |
| TyG (index) | 124.34 | 2 | 1.00E-27 |
| PP (mmHg) | 1.31 | 2 | 0.519 |
| LDL-C(mg/dL) | 10.73 | 2 | 0.00469 |
| WWI (cm/√kg) | 0.20 | 2 | 0.907 |

Restricted cubic spline (RCS) models with 4 knots (placed at the 5th, 35th, 65th, and 95th percentiles) were fitted for each continuous predictor in univariable logistic regression models in the development cohort (2016–2019). The nonlinearity of each predictor was tested by comparing the spline model with the corresponding linear-term model using a likelihood ratio test. The χ² statistic, degrees of freedom (df), and P value for nonlinearity are reported. A small P value indicates evidence against the linearity assumption.

**Table S22 Multivariable spline sensitivity analysis (TyG & AIP with restricted cubic splines)**

| **Model** | **AUC** | **Brier score** | **CITL** | **Calibration slope** |
| --- | --- | --- | --- | --- |
| Main elastic-net (linear terms) | 0.7465 | 0.0943 | -0.1828 | 1.2009 |
| Sensitivity elastic-net (spline TyG + AIP) | 0.7577 | 0.0934 | -0.0513 | 1.2970 |

Model performance was evaluated in the 2021 temporal validation cohort. Discrimination was assessed using the area under the receiver operating characteristic curve (AUC), overall prediction error using the Brier score, and calibration using the calibration-in-the-large (CITL) and calibration slope. Higher AUC and lower Brier score indicate better discrimination and overall accuracy, respectively; CITL close to 0 and slope close to 1 indicate good calibration.
